# Golgi fragmentation – One of the earliest organelle phenotypes in Alzheimer’s disease neurons

**DOI:** 10.3389/fnins.2023.1120086

**Published:** 2023-02-16

**Authors:** Henriette Haukedal, Giulia I. Corsi, Veerendra P. Gadekar, Nadezhda T. Doncheva, Shekhar Kedia, Noortje de Haan, Abinaya Chandrasekaran, Pia Jensen, Pernille Schiønning, Sarah Vallin, Frederik Ravnkilde Marlet, Anna Poon, Carlota Pires, Fawzi Khoder Agha, Hans H. Wandall, Susanna Cirera, Anja Hviid Simonsen, Troels Tolstrup Nielsen, Jørgen Erik Nielsen, Poul Hyttel, Ravi Muddashetty, Blanca I. Aldana, Jan Gorodkin, Deepak Nair, Morten Meyer, Martin Røssel Larsen, Kristine Freude

**Affiliations:** ^1^Department of Veterinary and Animal Sciences, Faculty of Health and Medical Sciences, University of Copenhagen, Frederiksberg, Denmark; ^2^Center for Non-coding RNA in Technology and Health, University of Copenhagen, Frederiksberg, Denmark; ^3^Novo Nordisk Foundation Center for Protein Research, University of Copenhagen, Copenhagen, Denmark; ^4^Centre for Neuroscience, Indian Institute of Science, Bengaluru, India; ^5^Copenhagen Center for Glycomics, Department of Cellular and Molecular Medicine, Faculty of Health and Medical Sciences, University of Copenhagen, Copenhagen, Denmark; ^6^Department of Biochemistry and Molecular Biology, University of Southern Denmark, Odense, Denmark; ^7^Department of Drug Design and Pharmacology, Faculty of Health and Medical Sciences, University of Copenhagen, Copenhagen, Denmark; ^8^Danish Dementia Research Centre, Department of Neurology, Neuroscience Centre, Copenhagen University Hospital - Rigshospitalet, Copenhagen, Denmark; ^9^Institute for Stem Cell Science and Regenerative Medicine, Bengaluru, India; ^10^Department of Neurobiology Research, Institute of Molecular Medicine, University of Southern Denmark, Odense, Denmark; ^11^Department of Neurology, Odense University Hospital, Odense, Denmark

**Keywords:** Alzheimer’s disease, hiPSC, neurons, Golgi fragmentation, disease modelling

## Abstract

Alzheimer’s disease (AD) is the most common cause of dementia, with no current cure. Consequently, alternative approaches focusing on early pathological events in specific neuronal populations, besides targeting the well-studied amyloid beta (Aβ) accumulations and Tau tangles, are needed. In this study, we have investigated disease phenotypes specific to glutamatergic forebrain neurons and mapped the timeline of their occurrence, by implementing familial and sporadic human induced pluripotent stem cell models as well as the 5xFAD mouse model. We recapitulated characteristic late AD phenotypes, such as increased Aβ secretion and Tau hyperphosphorylation, as well as previously well documented mitochondrial and synaptic deficits. Intriguingly, we identified Golgi fragmentation as one of the earliest AD phenotypes, indicating potential impairments in protein processing and post-translational modifications. Computational analysis of RNA sequencing data revealed differentially expressed genes involved in glycosylation and glycan patterns, whilst total glycan profiling revealed minor glycosylation differences. This indicates general robustness of glycosylation besides the observed fragmented morphology. Importantly, we identified that genetic variants in Sortilin-related receptor 1 (*SORL1*) associated with AD could aggravate the Golgi fragmentation and subsequent glycosylation changes. In summary, we identified Golgi fragmentation as one of the earliest disease phenotypes in AD neurons in various *in vivo* and *in vitro* complementary disease models, which can be exacerbated *via* additional risk variants in *SORL1*.

## Graphical Abstract

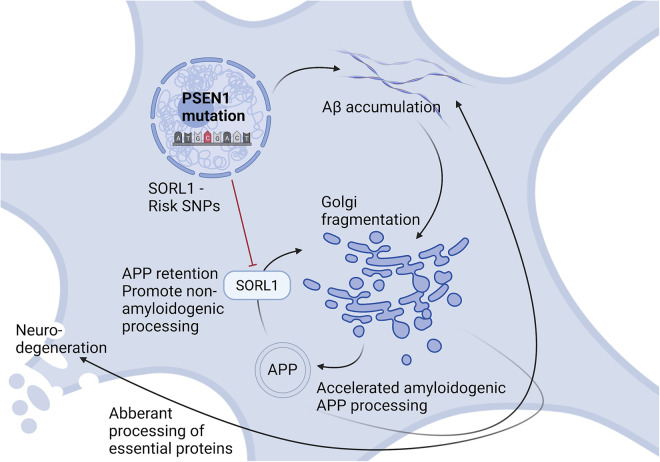

## 1. Introduction

Alzheimer’s disease (AD) is the most common cause of dementia, accounting for approximately 70% of all cases, with no available curative treatment (Alzheimer’s Association, 2022). Familial AD (fAD) presents as early-onset AD and is caused by mutations in *Presenilin 1*, *Presenilin 2* (*PSEN1*, *PSEN2*) or *Amyloid Precursor Protein (APP)* (Bekris et al., 2010). Although fAD is causative for only 1–5% of AD cases (Niikura et al., 2006), the same pathological hallmarks are shared between fAD and the more abundant multifactorial sporadic AD (sAD).

Most fAD cases carry mutations in *PSEN1*, a catalytic subunit of γ-secretase, responsible for the final cleavage of APP into amyloid beta (Aβ) peptides. Aβ accumulation leads to aggregation and build-up of extracellular Aβ plaques in patient brains. Together with intracellular neurofibrillary tangles (NFTs), caused by Tau hyperphosphorylation, these are considered classical pathological hallmarks of AD (Iqbal et al., 2005; Metaxas and Kempf, 2016; Selkoe and Hardy, 2016) and are characteristic in late stages of AD. However, AD pathogenesis initiates decades before these pathologies and symptoms appear, and precise disease mechanisms remain to be elucidated. Mitochondria, metabolic deficits and synaptic dysfunction have been widely studied in relation to AD, and it is evident that all of these are implicated in disease progression (Balaban et al., 2005; Moreira et al., 2010; Marsh and Alifragis, 2018; Liu et al., 2019). Nevertheless, the exact timeline and initial triggers of these neuronal phenotypes remain elusive, emphasizing the need to fully understand neuron-specific pathology to remedy the current lack of efficient AD treatments, so far solely focusing on counter-acting Aβ and Tau pathology.

A potential relevant cellular phenotype in AD is Golgi fragmentation, which has recently been reported in various neurodegenerative disorders (Martínez-Menárguez et al., 2019). The Golgi apparatus is the primary site of trafficking, processing, and sorting of most proteins and lipids, potentially linking Golgi fragmentation to abnormal post-translational processing. Alterations in Golgi structure or function can disrupt processing of AD-related molecules, which has been linked to both Aβ and Tau pathologies (Joshi et al., 2015). Moreover, altered trafficking of proteins and metabolites could affect synaptic function and overall neural health. In AD, disruptions of the Golgi stacks have been observed in post-mortem AD brains and transgenic mouse models (Baloyannis, 2014; Joshi et al., 2014; Antón-Fernández et al., 2017), and loss of Golgi ribbons or stack integrity is expected to affect membrane transport, glycosylation, and signaling networks (Li et al., 2019). These findings led us to investigate if Golgi fragmentation and altered glycosylation act as early events in AD pathogenesis, ultimately promoting neurodegeneration in human induced pluripotent stem cell (hiPSC) derived neuronal fAD models. We further extended our study including sAD hiPSC models, and the *in vivo* 5xFAD mouse model to investigate the universal relevance of this early disease pathology.

## 2. Materials and methods

### 2.1. hiPSC generation and cell culture

The hiPSC fAD cell lines were derived from a patient carrying the fAD-linked A79V *PSEN1* mutation (Li et al., 2016) and its gene corrected isogenic control A79V GC (Pires et al., 2016), a patient carrying the fAD-linked L150P *PSEN1* mutation (Tubsuwan et al., 2016) and its gene corrected isogenic control L150P GC (Poon et al., 2016), a healthy control [K3P53, (Rasmussen et al., 2014)] and a CRISPR/Cas9 gene edited knock-in *APP* Swedish fAD line [BioSweden, (Frederiksen et al., 2019)]. All fAD lines have previously been published (Supplementary Table S1A). The sAD cell lines have not been previously published but have been characterized and assessed for pluripotency markers and genomic integrity (Supplementary Figure 2). The hiPSCs were cultured in Essential 8 (E8) media (A1517001, Thermo Fisher Scientific, United States) on Matrigel-coated plates (TH Gayer, 7643022), and media was replaced every day. The cells were passaged every third day for approximately two weeks, before neural induction was initiated.

### 2.2. Neural induction and differentiation

Once hiPSC reached approximately 90% confluence, neural differentiation was performed according to a modified dual SMAD protocol (Shi et al., 2012). Neural induction was initiated by changing the media into neural induction media containing 50% DMEM/F12 (Thermo Fisher Scientific, 11330057), 50% advanced neurobasal medium (Thermo Fisher Scientific, 21103049), 1% N2 (Thermo Fisher Scientific, 17502048), 1% B27 without retinoic acid (Thermo Fisher Scientific, 1258010), 1% Glutamax™ (Thermo Fisher Scientific, 35050061), 1% non-essential amino acid (NEAA, Thermo Fisher Scientific, 11140-050), 0.1% Pen/Strep (Sigma-Aldrich, United States, P0781-100ML), supplemented with the inhibitors 10 μM SB431542 (SMAD inhibitor, SMS-gruppen, S1067) and 0.1 μM LDN193189 (Noggin analog, Sigma-Aldrich, SML0559). The cells were maintained in induction media for 12 days with daily media change. On day 12, a uniform neuroepithelial sheet appeared, and the neural progenitor cells (NPCs) were passaged with Accutase (Thermo Fisher Scientific, A1110501) into neural expansion media containing growth factors 10 ng/ml FGF2 (ProSpec, CYT-557) and 10 ng/ml EGF (ProSpec, CYT-217) instead of the inhibitors. NPCs were expanded and banked. Following expansion, NPCs were plated onto Poly-L-Ornithine (PLO, Sigma-Aldrich, P4957)/laminin (Sigma-Aldrich, L2020-1 mg) coated dishes with a seeding density of 50,000 cells/cm^2^, and terminal neural differentiation was performed in neural maturation media, supplemented with 50 μM db-cAMP (Sigma Aldrich, D0627-100 mg), 200 μM Ascorbic acid (Sigma Aldrich, A4403-100MG), 20 ng/ml BDNF (ProSpec, CYT-207) and 10 ng/ml GDNF (ProSpec, CYT-305). The maturation process was carried out for 5 weeks for MitoTracker™ and Golgi ICC analysis and 7 weeks for assessment of Aβ secretion and Tau phosphorylation as well as MitoTracker™, Golgi and synaptic evaluation, with partial media change every third day, before the neurons were fixed or harvested for further analyses.

### 2.3. Mesoscale assessment of Aβ peptide secretion

Secretion of Aβ peptides; Aβ38, Aβ40, and Aβ42, was assessed using the V-PLEX Plus Aβ Peptide Panel 1 Kit (MSD, 6E10), and the MESO QUICKPLEX SQ 120 Imager (MSD), connected to the DISCOVERY WORKBENCH 4.0 software, according to the manufacture’s protocol. The data was normalized to the RNA concentration of the individual samples, and data was obtained from three independent experiments.

### 2.4. Immunocytochemistry and confocal microscopy

For immunocytochemistry (ICC), neurons were plated and cultured on PLO/laminin-coated, double acid-treated coverslips for 5 weeks for MitoTracker™ and Golgi assessment and 7 weeks for neural verification, MitoTracker™, Golgi, and synaptic evaluation, fixed in 4% Paraformaldehyde (PFA) for 20 min at room temperature. Following, neurons were washed 3 × 5 min in PBS (Sigma, D8537). Neurons were then permeabilized with 0.2% Triton-X-100 solution for 20 min at room temperature, then blocked with 3% Bovine Serum Albumin (BSA, Sigma) for 30 min at room temperature. Neurons were incubated with primary antibodies (Supplementary Table S1B), diluted in 3% BSA, overnight at 4°C, washed with PBS 3 × 5 min at room temperature, and incubated with secondary antibodies (Supplementary Table S1B) for 1 h in the dark at room temperature. Another 3 × 5 min wash with PBS was performed, followed by DNA labeling with DAPI, diluted in PBS, for 7 min dark at room temperature. The neurons were then washed 3 × 5 min in PBS, before coverslips were mounted onto slides in DAKO fluorescence mounting solution and analyzed with the Leica confocal TCS SPE Microsystem implementing LAS × software. Obtained images were then processed in Fiji ImageJ 2.0.0-rc-65/1.51s. Data was obtained from three independent experiments.

### 2.5. Neurite outgrowth analysis

After 5 days of maturation, neurons were fixed in 4% PFA and ICC was performed with Beta-III-Tubulin (Tuj1, Millipore, MAB1637). Neurite length was measured and analyzed using Neurite Tracer in Fiji ImageJ (Pool et al., 2008), from three independent experiments.

### 2.6. MitoTracker™ assay

Neurons were plated on coverslips, cultured for seven weeks, then incubated with 50 nm MitoTracker™ Red CMXRos (Invitrogen/Molecular Probes, M7512) in DMEM/F12 for 15 min at 37°C. Following, the neurons were fixed in 4% PFA, permeabilized in 0.2% Triton X-100 in PBS, incubated with DAPI for DNA labeling, washed and then mounted onto slides in mounting media. Neurons from three independent experiments were imaged by confocal microscopy and analyzed in Fiji ImageJ. For detailed description see section: Immunocytochemistry and Confocal Microscopy.

### 2.7. Transmission electron microscopy

Neurons were cultured on coverslips, and after 7 weeks of maturation fixed with 3% Glutaraldehyde (Merck, 1042390250) in 0.1 M Na-phosphate buffer with pH 7.2 at 4°C for 1 h. The neurons were then embedded in 4% agar and cut into 1–2 mm^3^ blocks under a stereomicroscope, then washed with 0.1 Na-phosphate buffer, followed by post-fixation in 1% osmium tetroxide (EMS) in 0.1% Na-phosphate buffer for 1 h at room temperature. Washing with MilliQ water was performed, followed by a stepwise dehydration in ethanol with increasing concentration. Propylene oxide (Merck) was used as an intermediate allowing for infiltration with Epon (TAAB, T031). The following day, neurons were embedded in Epon, and cured at 60°C for 48 h. Semi thin sections (2 μm) were cut on an ultramicrotome with glass knives (Leica Ultracut, Leica Microsystems, Wetzlar, Germany), then contrasted with 1% Toluidine blue (Millipore, 1159300025) in 1% Borax (LabChem, LC117101). Ultra-thin sections (50–70 nm) were cut on the microtome with a diamond knife (Jumdi, 2 mm), and the sections were collected onto grids, and then stained with 2% uranyl acetate (PolyScience) and lead citrate (Reynolds, 1963). Imaging and analysis were performed “blinded” using a Philips CM100 transmission electron microscope with a Morada digital camera equipment and iTEM software system (Olympus).

Morphometry was used for quantitative evaluation of mitochondria, and five grids from each cell line were used for the analysis. A total of 15 spots were randomly chosen at low magnification and positions were stored according to their X and Y coordinates using FEI/Philips CompuStage. Images of each spot were captured using high magnification (19,000X), resulting in 75 images per cell line, and a total of 300 images. Three mitochondria categories were established based on the qualitative evaluation: Normal mitochondria with well-defined cristae throughout the organelle, cristaless mitochondria where these structures were lacking, and an additional category with mitochondria not fitting either of the two former categories (undefined). Mitochondria morphology and size were evaluated, and Fiji ImageJ was used for analysis by application of a grid to each image. Intersections were counted for each mitochondria category, as well as the cytoplasm and the number of mitochondria to calculate the relative mitochondria/cytoplasm ratio and the relative individual mitochondria area. Data was obtained from three independent experiments.

### 2.8. Western blot

Neurons were cultured for 7 weeks, washed with PBS, then lysed in M-PER mammalian protein extraction reagent (Thermo Fisher Scientific, 78501) with cOmplete™ Protease Inhibitor (Roche, 11873580001) and PhosSTOP™ phosphatase inhibitor (Roche, 04906845001). Protein content was determined using the Bradford Assay (Sigma, B6916). Separation of 10 μg protein was performed by NuPAGE™ Novex™ 4–12% Bis-Tris mini gel (Thermo Fisher Scientific, NP0335BOX), and immunoblotted with primary antibodies (Supplementary Table S1C) overnight at 4°C, followed by secondary antibody incubation (Supplementary Table S1C). The immunoblots were developed using Odyssey*^R^* Fc Imaging System (LI-COR) and analyzed using Image Studio version 5.2.5 and Excel. Expression levels of target proteins were normalized against housekeeping proteins (GAPDH or β-actin), and data was obtained from three independent experiments.

### 2.9. Amyloid beta treatment

To evaluate the implication of Aβ on the neurons, both the isogenic controls (A79V GC and L150P GC) and healthy control (K3P53) were treated with 2.5 μM Aβ peptide 1–42 (Sigma, PP69-0.25MG), incubated at 37°C for 24 h, then fixed and harvested for ICC and transmission electron microscopy (TEM). The analysis was performed in three individual experiments.

### 2.10. RNA extraction for bulk RNA sequencing

RNA was extracted from L150P and L150P GC as well as A79V and A79V GC neurons after 7 weeks of neural maturation, using the RNeasy^®^ Plus Mini Kit (Qiagen, The Netherlands, 74134), according to the manufacturer’s protocol, and the RNA quality was assessed using an Agilent 2100 Bioanalyzer system with RNA 6000 nano chip and reagents. Library preparation and sequencing (DNBseq, 2 × 100 nt, stranded paired-end) was done by an external provider for five independent replicates of fAD and isogenic control neurons (Beijing Genomics Institute, BGI, China).

### 2.11. Analysis of bulk RNA sequencing data

The computational analysis of the RNA sequencing data is described in detail in a separate bioinformatics publication (Corsi et al., 2022b). Briefly, reads were pre-processed with Cutadapt v1.18 (Martin, 2011) to trim low quality 3’ ends (min. Phred = 30), remove residual adapters, and filter out resulting reads shorter than 20 nt (pair_filter = “any”). Sequences matching to rRNAs in SILVA v119.1 (Quast et al., 2013) were removed with BBDuk v38.22.^1^ Genes were quantified from the reads pairs aligned with STAR v.2.6.1d (Dobin et al., 2013) on the human genome (hg38) using featureCounts (subread v.1.6.3, min. overlap 90 nt, min. fraction overlapping nt 90%, both aligned reads pairs non-chimeric) (Liao et al., 2014) provided with an extended set of annotations obtained by merging the GENCODE (Frankish et al., 2019) and the FANTOM-CAT gene models (Hon et al., 2017). Differential expression analysis was carried out using DESeq2 v1.22.2 (Love et al., 2014) and genes with Benjamini and Hochberg (1995) adjusted Wald test *p*-value < 0.05, absolute log2 fold change >1, and mean of normalized counts >10 were considered significant. The workflow includes RseQC v.4.0.0 (Wang et al., 2012) for the confirmation of the library strandness and the verification of the editing status at on-target and potential off-target sites with CRISPRroots v.1.2 (Corsi et al., 2022a). Potential off-targets identified by CRISPRroots as high-risk were validate by sequencing, and no off-target edit was found (Corsi et al., 2022b).

### 2.12. qPCR validation

RNA from L150P and L150P GC, A79V and A79V GC as well as BioSweden and K3P53 hiPSC derived neurons after seven weeks of neural maturation was extracted using the RNeasy^®^ Plus Mini Kit (Qiagen, 74134). cDNA was synthesized from 1 μg of total RNA, according to Promega ImProm-II™ Reverse Transcription System (Promega, United States, A3800). RNA was mixed with 1:3 oligoT: random primer (0.5 μg/μl), heated at 70°C for 5 min, then immediately put on ice for 5 min. Reverse transcription mix containing ImProm II buffer, dNTP mix (10 mm), Nuclease-free water, RNasin Ribonuclease inhibitor (40 u/μl), MgCl_2_ (2.5 mm) and ImProm II was added, following 5 min incubation at room temperature, then 1 h at 42°C. The enzyme was inactivated at 70°C for 15 min, and appropriate dilutions (1:5) of cDNA were prepared for further qPCR.

For qPCR analysis, nuclease-free water, QuantiFast SYBRGreen 2x (Qiagen), forward- and reverse primers (10 μM, Supplementary Table S1E) were mixed and added to each well of a qPCR plate, with a total of 8 μL per reaction and 2 μL of diluted cDNA was added. The qPCR was performed in triplicates, and a non-template control without cDNA was included for each target gene. The analysis was run on a LightCycler^®^ 480 real-time PCR system (Roche, Switzerland) with a total of 40 cycles, and the data was collected and processed using the Design and Analysis software 2.6.0. The data were normalized to *GAPDH* gene, and the analysis was performed in triplicates.

### 2.13. Proteomic assessment

For proteomic assessment, cell pellets from L150P and L150P GC as well as A79V and A79V GC neurons were collected using ice-cold PBS and a cell scraper. Following, proteomic assessment was performed by mass spectrometry, according to a previously published protocol (Bogetofte et al., 2019). Data was obtained from three independent replicates.

### 2.14. *N*- and *O*-glycan profiling by mass spectrometry

From L150P and L150P GC, A79V and A79V GC as well as BioSweden, K3P53 and Aβ-treated K3P53 hiPSC derived neurons, total cell lysate protein *N*- and *O*-glycans were released and analyzed by C18 nanoflow liquid chromatography (LC) coupled to mass spectrometry (MS) as described previously, with minor adaptations (de Haan et al., 2022). Cell pellets were resuspended at ∼2 × 10^4^ cells/μL in lysis buffer [50 mm Tris HCl, 100 mm NaCl and 1× cOmplete™ protease inhibitor (EDTA-free)] and loaded on a preconditioned PVDF membrane. *N*-glycans were released using 2 U PNGase F in 30 μL water, eluted and dried. Sialic acids were derivatized by ethyl esterification (α2,6-linked sialic acids) and subsequent ammonia amidation (α2,3-linked sialic acids) (Lageveen-Kammeijer et al., 2019) and purified by hydrophilic interaction liquid chromatography (HILIC) solid phase extraction (SPE) (Selman et al., 2011). Next, 50 μL 2-aminobenzamide (2-AB) reagent (500 mm 2-AB, 116 mm 2-methylpyridine borane complex (PB) in 45:45:10 metanol:water:acetic acid) was added and the samples were incubated 2.5 h at 50°C. The glycans were purified by HILIC SPE and eluted in 50 μL water. Ten microliters of the eluates were diluted in 10 μL water for MS analysis. *O*-glycans were released from the same samples on the PVDF membrane using 20% hydroxylamine and 20% 1,8-diazabicyclo(5.4.0)undec-7-ene (DBU) for 1 h at 37°C, and enriched by hydrazide beads, 2-AB labeled as described above, and purified by HILIC and porous graphitized carbon (PGC) SPE (de Haan et al., 2022). Samples were resolved in 20 μL water for MS analysis. For both the *N*- and *O*-glycan preparations of each sample, 2 μL was injected for nanoLC-MS/MS analysis, using a single analytical column setup. The analytical column was prepared using a PicoFrit Emitter (New Objectives, 75 μM inner diameter), packed with Reprosil-Pure-AQ C18 phase (Dr. Maisch, 1.9 μM particle size, 22–25 cm column length). The emitter was interfaced to an Orbitrap Fusion Lumos mass spectrometer (Thermo Fisher Scientific) *via* a nanoSpray Flex ion source. Samples were eluted in an 1 h method with a gradient from 3% to 32% of solvent B in 35 min, from 32% to 100% B in the next 10 min and 100% B for the last 15 min at 200 nl/min (solvent A: 0.1% formic acid in water; solvent B: 0.1% formic acid in 80% ACN). A precursor MS scan (m/z 200–1700, positive polarity) was acquired in the Orbitrap at a nominal resolution of 120,000, followed by Orbitrap higher-energy C-trap dissociation (HCD)-MS/MS at a nominal resolution of 50,000 of the 10 most abundant precursors in the MS spectrum (charge states 1 to 4). A minimum MS signal threshold of 30,000 was used to trigger data-dependent fragmentation events. HCD was performed with an energy of 27% ± 5%, applying a 20 s dynamic exclusion window. Data analysis and structural annotation were performed as described before (de Haan et al., 2022) using the Minora Feature Detector node in Thermo Proteome Discoverer 2.2.0.388 (Thermo Fisher Scientific Inc.), GlycoWorkbench 2.1 (build 146), (Damerell et al., 2012) Skyline 21.1.0.146 (ProteoWizard) (Adams et al., 2020) and the Thermo Xcalibur Qual browser 3.0.63. MS/MS spectra were manually assigned for each MS1 feature in at least one sample. *N*- and *O*- glycans were relatively quantified separately, by total area normalization. Derived traits were calculated based on specific glycosylation features, including for *N*-glycans the glycan type (paucimannose, oligomannose, complex or hybrid) and complex-type fucosylation (no or core and/or antenna), sialylation (no or α2,3- and/or α2,6-linked), bisection, LacdiNAc, galactosylation, and antenarity. For the *O*-glycans, the relative abundance of the *O*-glycan types was determined (*O*-GlacNAc, *O*-GalNAc, *O*-fucose, *O*-glycose or *O*-mannose), and specifically for the *O*-GalNAc glycans the relative abundance of the different cores (Tn, 1, 2, or 3) as well as the level of sialylation per core type. All values per cell type were represented as averages and standard deviations over three technical replicates.

### 2.15. Airyscan super-resolution microscopy and analysis

Neuro 2A (N2A) cells were transiently transfected with either *APP* Swedish- or wild type human APP (*hAPP*), using Lipofectamine 2000 (Thermo Fisher Scientific, 11668030) following a similar protocol as described previously (Kedia et al., 2020). Briefly, after incubation, diluted DNA and Lipofectamine were combined and incubated for 20 min at room temperature to establish DNA-Lipofectamine complexes. The complex (500 μL) was then added to the N2A cells, which were further incubated for 24-72 h at 37°C. Media was replaced after 6 h. Following transfection, the N2A cells were fixed and immunocytochemical labeling for GM130 and γ-adaptin (Supplementary Table S1B) was performed. The N2A cells were mounted with Prolong (Molecular Probes, cat. no. MAN0010261) for Airyscan super-resolution microscopy. Airyscan super-resolution microscopy was performed as described previously (Kedia et al., 2021). Airyscan images were obtained on Zeiss LSM 880 equipped with 32 array detectors for acquisition of super-resolution images. For image acquisition, 405, 488, and, 633 nm lasers were used. The illumination parameters like intensities, digital and analogue gain of the detectors, sampling of the images, emission window for each fluorescent channel and their corresponding pinhole sizes were maintained constant across acquisition paradigm. The raw images acquired using Airyscan mode were processed using Zeiss ZEN 3.4 (Blue) software to generate final super-resolution images. The reconstruction parameters were also kept constant throughout the samples. The morphological and biophysical traits of cis-/trans-Golgi were quantified on super-resolution images through MetaMorph software (Molecular Devices).

### 2.16. 5xFAD transgenic mice

To further validate our findings, male 5xFAD transgenic mice (*N* = 5) and male wild-type mice (*N* = 4) were sacrificed, and brains were dissected. The cortex and hippocampus were fixed in 3% Glutaraldehyde and embedded in Epon for TEM evaluation. For detailed description of TEM preparation and imaging, see the section TEM.

### 2.17. Statistical analysis and data availability

Statistical analyses were performed using GraphPad Prism (Version 9.2.0) with default options, and statistical significance was determined using a Student’s *t* test, multiple *t* test or two-way ANOVA with correction for multiple comparison. Data is presented as mean ± standard error of the mean (SEM) for all experiments with statistical significance *p < 0.05, ^**^p < 0.01, ^***^p < 0.001, and ^****^p < 0.0001. Gene differential expression was performed using DESeq2 (Version 1.22.2) (Love et al., 2014) using the Benjamini-Hochberg method to adjust Wald test *p*-values in multiple testing.

## 3. Results

### 3.1. Generation and characterization of AD neurons from human induced pluripotent stem cells

**FIGURE 1:**
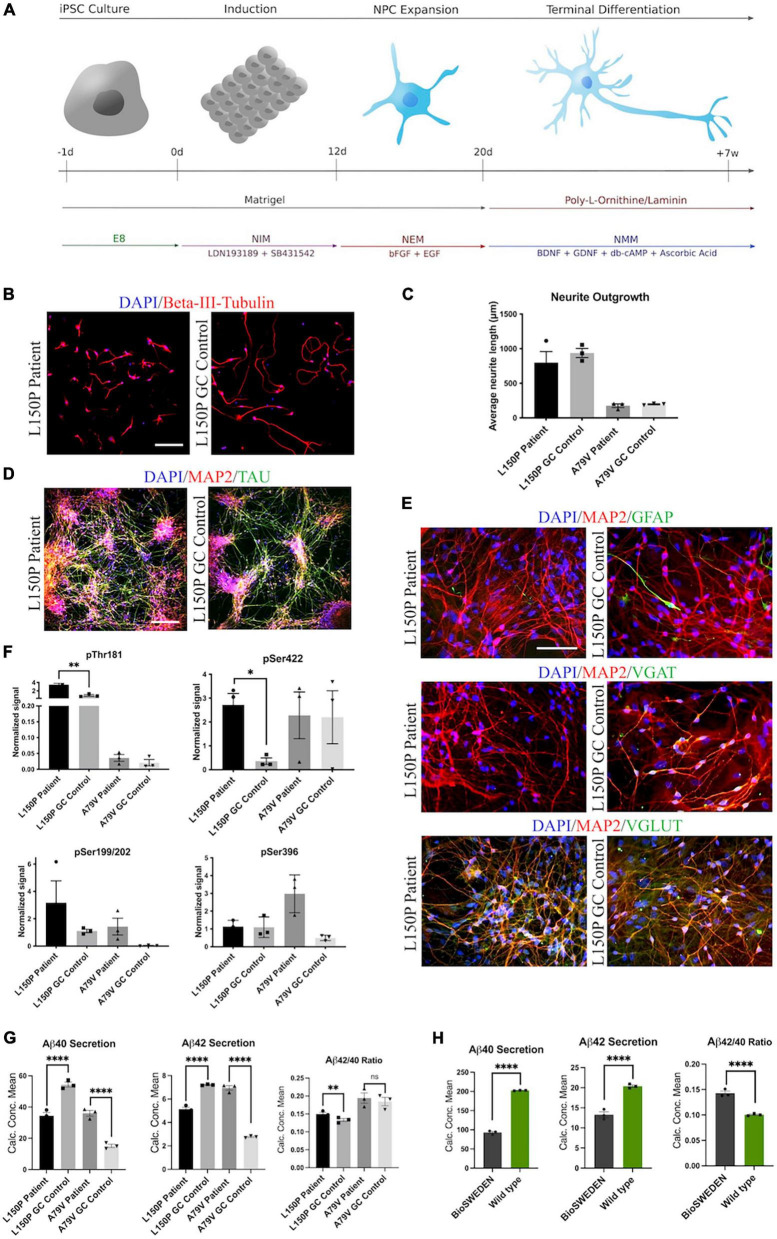
Generation and characterization of hiPSC derived neurons. **(A)** Schematic overview of the neural differentiation protocol. **(B)** Neurite outgrowth analysis *via* ICC expression of Beta-III-tubulin. Scale bar 100 μm. **(C)** Quantitative assessment of average neurite outgrowth. **(D)** Representative ICC images of neuronal markers MAP2 and TAU. Scale bar 100 μm. **(E)** Representative ICC images of MAP2, astrocytic marker GFAP, GABAergic neuron marker VGAT, and glutamatergic neuron marker VGLUT. Scale bar 50 μm. **(F)** Quantitative assessment of Tau phosphorylation (all four isoforms) - pThr181, pSer422, pSer199/202, and pSer396. **(G)** Quantitative assessment of Aβ40 and 42 secretion, and Aβ42/40 ratio in L150P and A79V hiPSC derived neurons. **(H)** Quantitative assessment Aβ40 and 42 secretion, and Aβ42/40 ratio in K3P53 and BioSweden hiPSC derived neurons. Results are displayed as mean ± standard error of the mean (SEM) from three replicates. Significance levels are indicated by *p < 0.05, ^**^p < 0.01, and ^*⁣*⁣**^p < 0.0001.

Neurons were differentiated from hiPSCs derived from two patients carrying fAD-linked *PSEN1* mutations and their respective isogenic controls corrected *via* CRISPR/Cas9 gene editing: L150P and L150P GC (gene corrected) (Poon et al., 2016; Tubsuwan et al., 2016), A79V and A79V GC (Li et al., 2016; Pires et al., 2016), as well as a healthy control- (K3P53) (Rasmussen et al., 2014) and a CRISPR/Cas9 knock-in hiPSC line carrying the Swedish *APP* mutation (BioSweden) (Frederiksen et al., 2019). fAD- and control-hiPSCs were successfully differentiated into cortical, glutamatergic forebrain neurons (Shi et al., 2012) (Figure 1A). Neurite outgrowth analysis revealed a non-significant tendency toward reduced neurite length in the AD lines compared to their respective isogenic controls after 1 week of differentiation (Figures 1B, C). Neural outgrowth capacity was initially lower for A79V and A79V GC, potentially indicating innate differences between the two hiPSC lines during the early stages of differentiation. However, all hiPSC derived neurons displayed comparable differentiation potential and maturity levels at week seven. This was assessed *via* expression of microtubule associated protein 2 (MAP2) and Tau (Figure 1D). Astrocyte content in our cultures ranged from 5% to 10%, detected by Glial Fibrillary Acidic Protein (GFAP) expression (Figure 1E). Most of the cultured neurons were glutamatergic neurons (VGLUT) with few GABAergic neurons (VGAT) (Figure 1E and Supplementary Figure 1).

### 3.2. AD neurons display amyloid beta- and tau pathologies

Western blot (WB) analyses revealed significantly increased levels of phosphorylated Tau (p-Tau) isoforms pThr181 and pSer422 in the L150P neurons. A similar trend for pSer199/S202 was observed, but no difference was detected for pSer396. A79V demonstrated similar tendencies but showed no difference in pSer422 levels (Figure 1F). Mesoscale V-PLEX assessment of Aβ peptides 1–40 and 1–42 revealed a significant increase in Aβ42/40 ratio in L150P neurons, likely due to a substantial decrease in Aβ40. This is in line with other *in vitro* studies showing that fAD-linked mutations in *PSEN1* can lead to reduced levels of individual Aβ peptides, with particularly low or even undetectable levels of Aβ40 (Sun et al., 2017). The increase in Aβ42/40 ratio was not significant in the A79V neurons; however, up-regulated secretion of both Aβ40 and Aβ42 was detected (Figure 1G), which aligns with other studies in different model systems of the A79V mutation (Kumar-Singh et al., 2006; Kauwe et al., 2007; Sun et al., 2017). Recapitulation of characteristic Aβ and Tau pathologies in our hiPSC derived cell models validates their relevance as *in vitro* AD models. Moreover, increased Aβ42/40 ratio was detected in BioSweden neurons, indicating that other fAD-linked mutations result in similar neuronal phenotypes (Figure 1H).

### 3.3. AD neurons display abnormal mitochondria morphology and distribution

**FIGURE 2:**
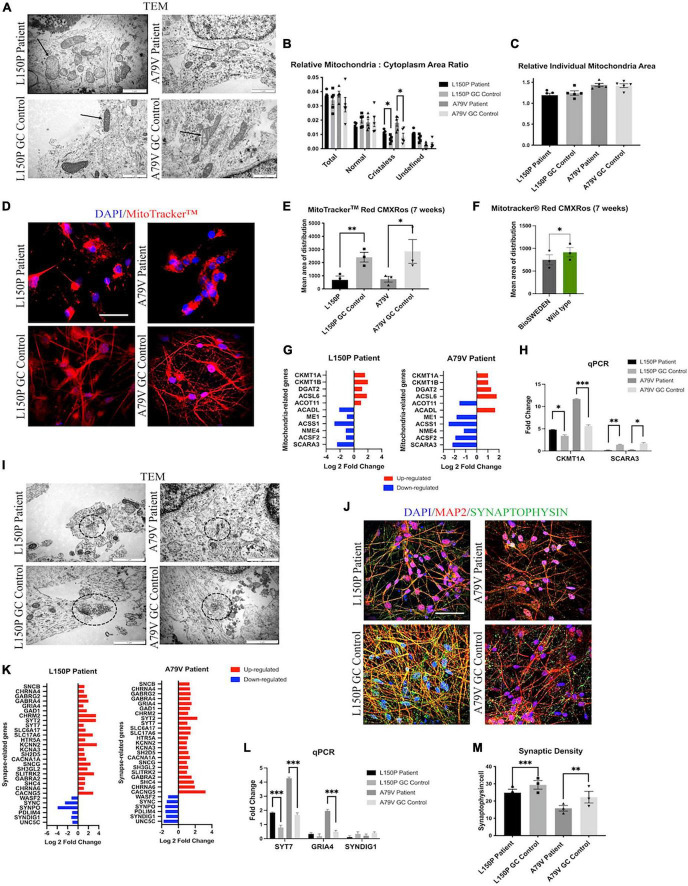
fAD neurons display mitochondria and synaptic deficits. **(A)** TEM evaluation of mitochondria (indicated by arrows) ultrastructure. Scale bar 1 μm. **(B)** Quantitative TEM morphometry assessment of relative mitochondria to cytoplasm ratio. **(C)** Quantitative TEM morphometry evaluation of relative individual mitochondria area. **(D)** Representative ICC images of MitoTracker™ analysis. Scale bar 50 μm. **(E)** Quantitative assessment of MitoTracker™ analysis showing mean area of distribution in L150P and A79V hiPSC derived neurons. **(F)** Quantitative assessment of MitoTracker™ analysis showing mean area of distribution in K3P53 and BioSweden hiPSC derived neurons. **(G)** Overview of top significantly differentially expressed genes associated with mitochondria. **(H)** qPCR validation of key differentially expressed genes associated with mitochondria identified by RNA-seq analysis. **(I)** TEM evaluation of synapses and synaptic vesicles (circled). Scale bar 1 μm. **(J)** Representative ICC images of synaptophysin expression. Scale bar 50 μM. **(K)** Overview of top significantly differentially expressed genes associated with synapses. Adjusted p-value is displayed in Supplementary Table 2C. **(L)** qPCR validation of key differentially expressed genes associated with synapses identified by RNA-seq analysis. **(M)** Quantitative assessment of synaptophysin expression. Results are displayed as mean ± standard error of the mean (SEM) from three replicates. Significance levels are indicated by *p < 0.05, ^**^p < 0.01, ^***^p < 0.001.

Next, we assessed the mitochondria ultrastructure in fAD and control neurons using TEM. Here, we demonstrated abnormal cristae-lacking organelle morphology (Figure 2A) and a significant increase of relative cristae-less mitochondria to cytoplasm ratio in both L150P and A79V neurons (Figure 2B). The relative individual mitochondria area was unaltered, indicating increased number of cristae-less organelles, and not mitochondria size (Figure 2C). TEM analysis revealed a perinuclear mitochondria accumulation in fAD neurons. MitoTracker™ confirmed a disrupted mitochondria distribution with a significant reduction in mean area of distribution in both L150P and A79V (Figures 2D, E). Rescue of mitochondria phenotypes in our isogenic controls shows that our findings can be attributed to the *PSEN1* mutations. The same distribution pattern was demonstrated in BioSweden neurons, indicating that mitochondria defects are equally relevant for both *PSEN1* and *APP* fAD-linked mutations (Figure 2F). Aberrant mitochondria morphology suggests impaired mitochondria function since the ATP production *via* electron transport chain takes place within the inner membrane, which depends on an intact membrane and correct cristae folding (Afzal et al., 2021). Abnormalities in energy production and metabolism were verified by computational analysis of RNA sequencing, which revealed differentially expressed genes related to mitochondria and oxidative stress, further validated by qPCR (Figures 2G, H), and supported by proteomics (Supplementary Table S2C). *CKMT1*, central to metabolism and energy transduction, was significantly increased in both L150P and A79V neurons (Figure 2G and Supplementary Table S2C). Such upregulation is commonly seen in conditions with compromised energy state (Schlattner et al., 2006), and might reflect a compensatory mechanism in response to impaired mitochondria function. Moreover, downregulation of *SCARA3*, normally protecting against reactive oxygen species (ROS), was detected in both L150P and A79V neurons (Figure 2G and Supplementary Table 2SC), indicating an impaired ability to handle oxidative stress and causing excessive neurotoxic effects (Peng et al., 2021). Combined, these findings validate mitochondria dysfunction early-on in AD pathogenesis in our seven-week fAD neurons, which are supported by previously described mitochondria phenotypes in the 5 × FAD mouse model (Andersen et al., 2021).

### 3.4. AD neurons display reduced synaptic density

Transmission electron microscopy (TEM) assessment further revealed a reduced number of synapses, which were identified by electron dense regions at both the pre- and post-synaptic terminal, as well as synaptic vesicles in L150P and A79V neurons (Figure 2I). Together with reduced synaptophysin expression identified *via* ICC analysis (Figures 2J, M), these findings indicate reduced synaptic density. Interestingly, transcriptome analysis revealed mainly upregulation of synapse-related genes (Figure 2K), validated by qPCR (Figure 2L) and proteomics analysis (Supplementary Table S2C), suggesting altered synaptic function. Such upregulation has been demonstrated in mild cognitive impairment (MCI) and early AD, potentially acting as a compensatory mechanism for rebalancing synaptic transmission in early-stage AD (Berchtold et al., 2014). The fact that our *in vitro* cell models display both mitochondria and synaptic deficits, in addition to Aβ and Tau pathology, further stresses their potential in AD modeling, to better understand early cell-type specific disease perturbations.

### 3.5. AD neurons display Golgi fragmentation

**FIGURE 3:**
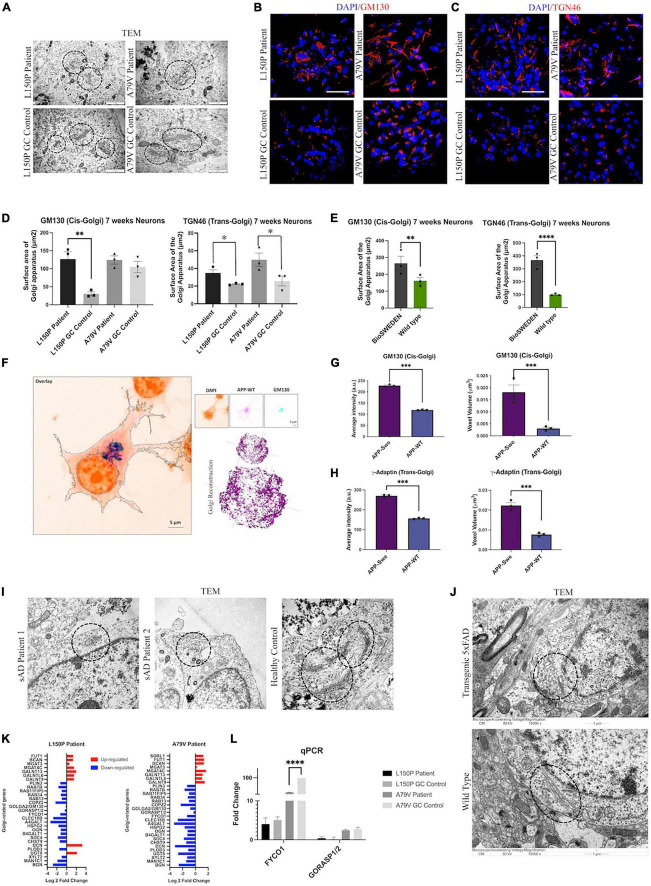
Golgi fragmentation is a universal phenotype in AD. **(A)** TEM evaluation of Golgi morphology (circled). Scale bar 1 μm. **(B)** Representative ICC images of cis-Golgi marker GM130. Scale bar 50 μm. **(C)** Representative ICC images of trans-Golgi marker TGN46. Scale bar 50 μm. **(D)** Quantitative assessment of cis- and trans-Golgi surface area in L150P and A79V hiPSC derived neurons. **(E)** Quantitative assessment of cis- and trans-Golgi surface area in K3P53 and BioSweden hiPSC derived neurons. **(F)** Representative images of cis-Golgi marker GM130 in APP-WT/Swe transfected N2A cells obtained using Airyscan super resolution microscopy. Scale bar 5 μm. **(G)** Quantitative assessment of biophysical (intensity) and morphological (voxel volume) traits of cis-Golgi in APP-WT/Swe transfected N2A cells. **(H)** Quantification of biophysical (intensity) and morphological (volume) traits of trans-Golgi in APP-WT/Swe transfected N2A cells. **(I)** TEM evaluation of dilated Golgi morphology (circled) in hiPSC derived neurons derived from sporadic AD (sAD) patients. Scale bar 1 μm. **(J)** TEM evaluation of Golgi fragmentation (circled) in a transgenic 5xFAD mouse model. **(K)** Top significantly differentially expressed genes associated with the Golgi apparatus. **(L)** qPCR validation of key differentially expressed -Golgi-associated genes identified by RNA-seq analysis. Results are displayed as mean ± standard error of the mean (SEM) from three replicates. Significance levels are indicated by *p < 0.05, ^**^p < 0.01, ^***^p < 0.001, and ^****^p < 0.0001.

Since Golgi fragmentation has been identified in neurodegenerative disorders (Martínez-Menárguez et al., 2019), we next investigated Golgi organization and function in L150P and A79V neurons. TEM analysis revealed an abnormal Golgi morphology with dilated cisternae and shortened Golgi stacks (Figure 3A). ICC analysis confirmed a disorganized Golgi pattern in L150P and A79V, compared to a centered Golgi at one pole in the perinuclear region in the isogenic controls, both for the cis- (GM130) (Figure 3B), and trans-Golgi (TGN46) (Figure 3C), indicating a total fragmentation, with an increase in cis- and trans-Golgi surface area (Figure 3D). This was particularly prominent for the trans-Golgi, with significant changes in both mutations at seven weeks. Our findings ware mirrored in BioSweden neurons (Figure 3E), demonstrating Golgi fragmentation as a characteristic phenotype common for both *APP* and *PSEN1* fAD mutations. Moreover, Golgi abnormalities were consistent and present in other model systems. Multicolor Airyscan super-resolution imaging of N2A cells, transfected with *hAPP* Swedish, confirmed cis- (GM130) and trans-Golgi (γ-adaptin) fragmentation, with significant increase in average intensity and organelle volume (Figures 3F–H). Moreover, Golgi fragmentation was present in two independent male and female sAD hiPSC derived neuronal models (Figure 3I). Likewise, Golgi fragmentation was demonstrated in the 5 × FAD transgenic mouse model (Figure 3J). We thus validated our findings in multiple fAD and sAD *in vitro*, as well as fAD *in vivo* models, indicating Golgi fragmentation as a universal early event in AD pathology.

Differential expression of Golgi-related genes in L150P and A79V neurons (Figure 3K), validated by qPCR (Figure 3L), further supported Golgi abnormalities. This included *GM130* and *GORASP*, involved in organization, assembly, and vesicle fusion, and *FYCO1*, containing a Golgi dynamics domain facilitating interactions and sorting. Additional differentially expressed genes related to glycosylation, which could indicate impaired Golgi function, include *GALNTs*, *MAN1C1*, and *MGATs* (Supplementary Table S2C).

### 3.6. Golgi fragmentation is an early trigger in AD pathogenesis

**FIGURE 4:**
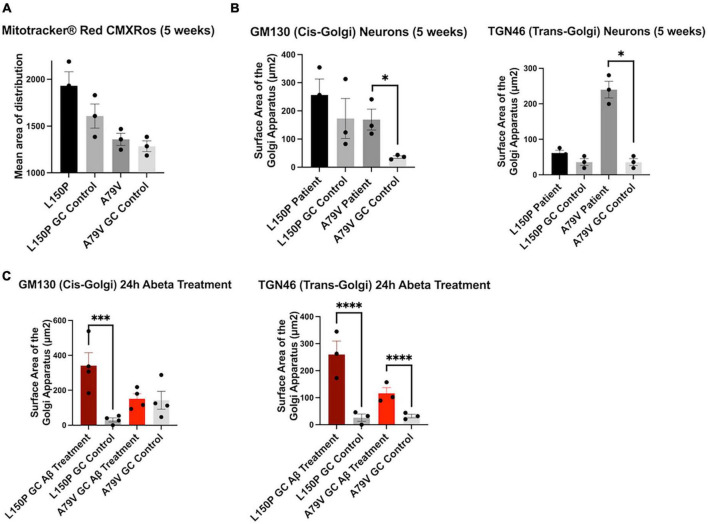
Golgi fragmentation is an early event in AD pathogenesis, triggered by Aβ42 peptides. **(A)** Quantitative assessment of MitoTracker™, mean area of distribution, in five-week L150P and A79V hiPSC derived neurons. **(B)** Quantitative assessment of cis- and trans-Golgi surface area in five-week L150P and A79V hiPSC derived neurons. **(C)** Quantitative assessment of cis- and trans-Golgi surface area in isogenic controls, following a 24-hour Aβ treatment. Results are displayed as mean ± standard error of the mean (SEM) from three replicates. Significance levels are indicated by *p < 0.05, ^***^p < 0.001, and ^****^p < 0.0001.

To investigate the initial triggers and timeline of AD pathogenesis we assessed our hiPSC derived neurons at both 5 and 7 weeks during the terminal differentiation phase. At week five, the mitochondria distribution remained intact (Figure 4A), whilst Golgi fragmentation was clearly present and particularly prominent in A79V (Figure 4B). Interestingly, the cis-Golgi surface area was significantly increased in five-week A79V neurons, opposed to the seven-week neurons, suggesting a compensatory mechanism that could contribute to maintain Golgi structure and function. Importantly, our results suggest that Golgi fragmentation is one of the earliest neuronal AD events, preceding mitochondria deficits.

### 3.7. Golgi fragmentation can be induced by amyloid beta peptide accumulation

Aβ-mediated phosphorylation of the Golgi-matrix protein GRASP65 has been indicated as a trigger of Golgi fragmentation (Joshi et al., 2014). Additionally, Golgi organization is dependent on an intact cytoskeleton, and Tau hyperphosphorylation might exacerbate the disorganization. Fragmentation can lead to a vicious cycle enhancing Aβ and Tau pathologies, ultimately promoting neurodegeneration (Joshi et al., 2014, 2015). To confirm if increased Aβ is the mechanism underlying the observed Golgi fragmentation, we treated seven-week isogenic control neurons with Aβ42 peptide for 24 h. Strikingly, this treatment induced fragmentation of both cis- and trans-Golgi, mirroring the seven-week fAD neurons (Figure 4C), clearly indicating that Aβ42 is a trigger of fragmentation. The potential contribution of Tau phosphorylation is highlighted by the fact that L150P neurons demonstrated significant Tau hyperphosphorylation and consequently displayed a more profound fragmentation of the cis-Golgi compartment.

### 3.8. Golgi fragmentation is not directly affecting glycosylation processing

**FIGURE 5:**
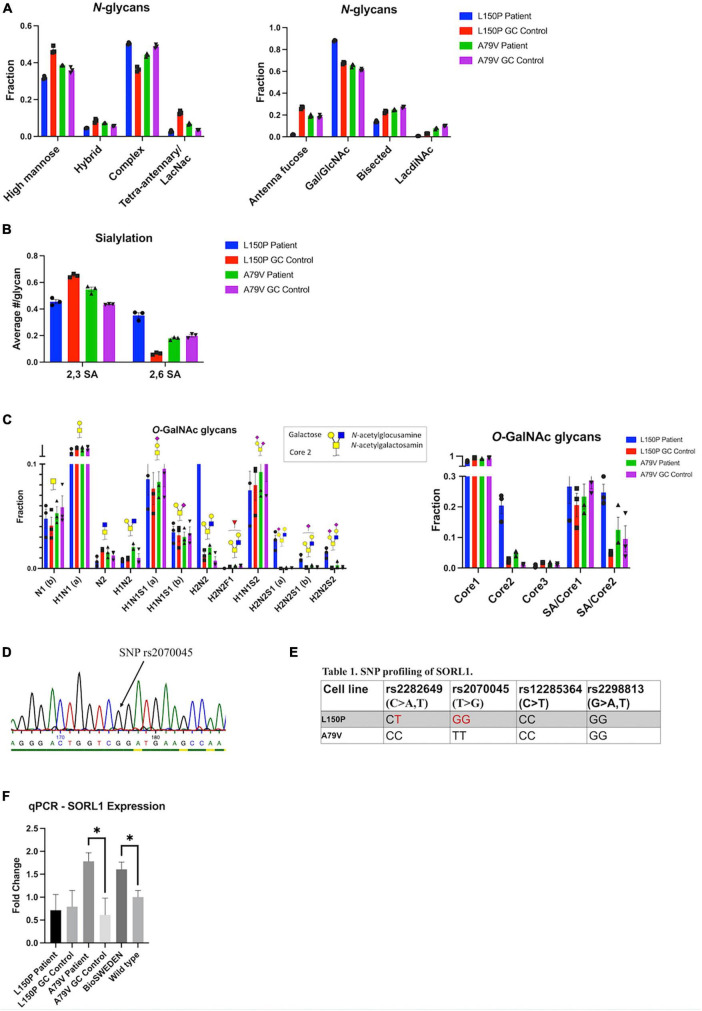
Golgi fragmentation is not directly affecting Golgi function, which is likely linked to multiple mechanisms. **(A)** *N*-glycan profiling. **(B)** *N-*glycan sialylation pattern. **(C)** *O*-glycan profiling. **(D)** Example of SNP profiling *via* Sanger sequencing. **(E)** SNP profiling of the *SORL1* gene. **(F)** qPCR validation of *SORL1* expression. Results are displayed as mean ± standard error of the mean (SEM) from three replicates. Significance levels are indicated by *p < 0.05.

The observed Golgi fragmentation together with changes in genetic expression suggests compromised Golgi function. Therefore, we investigated glycosylation *via* total glycan profiling of our hiPSC derived neurons. Overall Golgi fragmentation appeared to have little impact, indicating a robust glycosylation machinery, and no consistent changes were observed in *N*-glycan profile for L150P and A79V. However, a few alterations could be observed within the L150P neurons, including a tendency of reduced high-mannose levels, low antenna fucosylation, bisecting GlcNAc, and LacdiNAc levels, increased antenna galactosylation (Figure 5A), as well as a sialic acid (SA) linkage shift from 2,3 SA to 2,6 SA (Figure 5B). The *N*-glycan profile of the A79V, as well as the BioSweden (Supplementary Figure 3), remained unchanged. A similar trend was observed in the *O*-glycan profile. L150P deviated slightly, showing an altered fucosylation pattern, displayed as absent H2N2F1 (Figure 5C), similar to the changes in antenna fucose *N*-glycans. These are likely regulated by the same enzymes, related to terminal glycosylation. Interestingly, L150P and A79V showed a comparable tendency toward increased core 2 *O*-GalNAc glycans (Figure 5C), which could imply that some stages of complex glycosylation could be affected by Golgi fragmentation. Taken together, the glycan alterations observed in the L150P neurons are likely to be disease-associated, but not exclusively linked to *PSEN1* mutations or the Golgi fragmentation.

### 3.9. Multiple mechanisms underlie golgi function

The finding of only minor glycan changes in A79V compared to L150P neurons suggests that other underlying mechanisms could maintain the function of the Golgi, despite fragmentation. Sortilin-related receptor 1 (*SORL1*) encodes a protein directly involved in APP processing, and mediates retrograde transport of APP between the trans-Golgi network (TGN) and early endosomes (Yin et al., 2015). Both genetic and functional alterations of *SORL1* has been linked to AD (Rogaeva et al., 2007; Rovelet-Lecrux et al., 2021), and single nucleotide polymorphisms (SNPs) in *SORL1* can confer increased risk of developing AD. Wild-type *SORL1* is suggested to have a protective effect (Rogaeva et al., 2007; Rovelet-Lecrux et al., 2021). Therefore, we assessed the genetic variants of *SORL1* in L150P and A79V hiPSC, *via* Sanger sequencing (Figure 5D) of the genomic locations within the *SORL1* protein-coding region, harboring four SNP sites correlated to AD risk: rs2282649, rs2070045, rs12285364, and rs2298813. Strikingly, we identified the presence of two of these SNP variants in L150P (rs2282649 heterozygote and rs2070045 homozygote), which might contribute to the functional Golgi alteration, causing altered protein trafficking within the trans-Golgi. No *SORL1*-linked SNPs were identified in the A79V (Figure 5E), wild-type K3P53 or knock-in BioSweden hiPSCs. Previous studies have reported decreased *SORL1* expression in AD (Dodson et al., 2008). Instead, A79V and BioSweden neurons showed upregulated *SORL1* expression (Figure 5F). This suggests a beneficial compensatory mechanism that retain Golgi function, which could be characteristic for early stages of AD.

## 4. Discussion

Mutations in *PSEN1* are the most common causes of fAD, but their precise function has yet to be fully elucidated. Here, we investigated phenotypes related to two fAD linked *PSEN1* mutations, as well as the *APP* Swedish mutation, in neurons derived from hiPSC. These models recapitulate characteristic disease hallmarks such as Aβ, Tau, mitochondria, and synaptic pathology (Yu and Lu, 2012; García-Escudero et al., 2013). More recently, Golgi fragmentation has been identified in AD models, including post-mortem AD brains (Joshi et al., 2015; Antón-Fernández et al., 2017). To date, it was not clear if mitochondria dysfunction precedes Golgi fragmentation or vice versa. Here, we report that Golgi fragmentation is evident prior to mitochondria deficits in our models, placing it as one of the initial events in AD neural pathology. Importantly, we show that fragmentation is consistent in both fAD and sAD, and throughout multiple *in vitro* and *in vivo* model systems, independent of the underlying mutations, reinforcing the relevance of our findings for AD in general. We further indicate that Aβ42 peptides can act as a triggering mechanism, suggesting a timeline where Aβ accumulation is sufficient to induce Golgi fragmentation, followed by mitochondria and synaptic dysfunction.

Glycosylation, which largely takes place in the Golgi, is the most common form of post-translational modification, and glycan changes have been identified in AD (Haukedal and Freude, 2021). To evaluate if Golgi function was impaired in a broader sense, we investigated the glycan pattern in our fAD neurons. Surprisingly, Golgi fragmentation had little impact on glycosylation, indicating a certain robustness of the glycosylation system. Remarkably, some changes in glycan patterns were evident for the L150P neurons, both within the *N*- and *O*-glycan profiles, revealing that the functional outcome of Golgi fragmentation is dependent on the underlying genetic profile of affected individuals and not only on mutations in *PSEN1* or *APP*. The glycan alterations found in L150P neurons mainly affected the final decoration of the sugar structures, which largely takes place in the trans-Golgi. There was no significant concordance between the glycogene candidates identified *via* RNA sequencing analysis and the global neuron glycosylation patterns. This suggests that the glycosylation in this study is not regulated at the level of the glycogene expression but rather on enzyme localization and carrier glycoprotein production. Such discrepancies between RNA networks and proteome are not uncommon and have previously been described in AD brains (Johnson et al., 2022). Importantly, fragmentation was more prominent in the L150P neurons, whilst the cis-Golgi was not significantly affected in A79V neurons, potentially reflecting the intact *N-*glycan profile. Reduced levels of high-mannose in L150P neurons supports this theory, as mannosidase activity occurs in the cis-Golgi (Velasco et al., 1993). Golgi fragmentation has been suggested to accelerate protein trafficking through increased surface area for vesicle budding and altered protein sorting (Joshi et al., 2014). Cis-Golgi fragmentation could hence contribute to increased mannosidase activity, explaining the reduced high-mannose levels only evident in L150P. Moreover, the *O*-glycan pattern in L150P neurons, which deviates slightly from the other lines, might explain the significant changes we observe in its p-Tau levels. Hence, the mainly intact *O*-glycosylation pattern in the A79V neurons could result in non-significant changes in Tau phosphorylation.

The only consistent trend in glycan pattern was upregulation of the complex *O*-glycan structure core 2, one of the processes that mainly occurs in the trans-Golgi. The mutually significant fragmentation of this compartment could thus underlie these effects. As hypothesized, if fragmentation enhances protein processing through increased vesicle budding and impaired protein sorting, it could alter the localization of proteins and their respective processing enzymes and fail to sort them into separate compartments, thus accelerating the post-translational processing, ultimately causing altered glycan patterns. However, the findings of a robust glycosylation process in A79V, BioSweden as well as Aβ-treated control neurons, suggest that although Aβ is sufficient to trigger Golgi fragmentation, multiple mechanisms might underlie Golgi function, and prolonged fragmentation might be needed to ultimately induce a functional effect. Moreover, although Golgi fragmentation had no direct or exclusive impact on overall glycosylation, other post-translational modification processes could potentially be affected.

Golgi fragmentation was dependent on the individual genetic profile, suggesting involvement of multiple mechanisms. *SORL1*, which is both genetically and functionally linked to AD risk, retains APP in the TGN, and can potentially exert a protective effect against Aβ toxicity (Yin et al., 2015). SORL1 and APP co-localize in the Golgi and endosomal compartments. Neural overexpression of *SORL1* leads to redistribution of APP to the Golgi and reduced amyloidogenic processing, whereas *SORL1* depletion causes increased Aβ production (Andersen et al., 2005). The extracellular plasma membrane has been considered the major site for α-secretase activity. However, recent studies in primary neurons have demonstrated α-secretase activity in the TGN, with increased levels of C83 (α-CTF) compared to C99 (β-CTF) following APP accumulation (Tan and Gleeson, 2019). This indicates TGN as a major additional site for α-secretase processing. *SORL1*-mediated APP retention in the TGN could thus be a protective mechanism. Mutant *SORL1* maintain APP-binding activity, but in contrary to wild-type *SORL1* leads to misdirected APP trafficking into non-Golgi compartments that in turn increases Aβ production (Fjorback et al., 2012).

We identified several risk-associated SNPs in *SORL1* in our L150P hiPSC, which could contribute to the observed altered Golgi function, absent in the other lines. Interestingly, *SORL1* expression was upregulated in both A79V and BioSweden neurons, in contrary to the decreased levels commonly seen in AD patients. These findings suggest a possible compensatory mechanism that might influence the glycosylation process and could potentially be correlated to the differences in Aβ peptides secretion. The Aβ42/40 ratio, which was increased in L150P, is directly correlated to Tau pathology (Kwak et al., 2020). On the contrary, A79V neurons displayed increased levels of individual Aβ40 and 42 levels, possibly linked to the APOE ε4/4 profile of this patient, suggested to increase Aβ production (Ye et al., 2005). Our observations further indicate a compensatory mechanism of *SORL1* that counteracts the increase in Aβ levels early-on in A79V neurons, eventually lowering the impact on perturbations of the Golgi. This is in line with the fragmentation pattern observed for the cis-Golgi, which is significant in five-week A79V neurons but restored at 7 weeks.

*Amyloid Precursor Protein (APP)* and *SORL1* contain multiple glycosylation sites and undergo processing within the Golgi. *SORL1*-mediated APP retention in the TGN potentially allows for proper processing, despite the compromised structure. Changes in glycosylation pattern of key AD-related molecules such as APP, Tau, BACE1, and APOE have previously been identified (Vanoni et al., 2008; Kizuka et al., 2016, 2017; Flowers et al., 2020; Losev et al., 2020). In this study we focused on global glycan changes, and the analyses account for the total abundance of neuronal glycan patterns and not individual proteins or the cellular localization. While powerful in giving an overall overview, this approach might miss more subtle changes. The global glycosylation effects observed could be driven by altered abundance of specific carrier glycoproteins. Alternatively, multiple proteins could be affected, but the functional relevance of these changes might differ for individual proteins, such as APP and *SORL1*.

Sortilin-related receptor 1 (*SORL1*) has been shown to interact with APOE, especially the ε4 variant. Reduced levels of *SORL1* have been observed in neural stem cells from APOE ε4/4 carriers (Zollo et al., 2017), whereas increased levels have been detected in cerebrospinal fluid (CSF) of AD patients (Ikeuchi et al., 2010). A79V neurons carry APOE ε4/4 and BioSweden carry APOE ε3/4. Based on our findings we propose that the presence of APOE ε4 correlates with upregulation of *SORL1* expression as a compensatory mechanism. Hence, we observed the opposite in L150P neurons carrying APOE ε2/3, displaying no upregulation of *SORL1* expression. *SORL1* overexpression has been shown to increase Aβ uptake in an APOE isoform-dependent manner, and more efficiently in the presence of the APOE ε4 isoform (Louwersheimer et al., 2017). Dysfunctional *SORL1* (caused by SNP variants) can lead to impaired binding to APP and Aβ. This could in turn alter APP trafficking, reduce *SORL1*-mediated retention in the TGN, and thus increase Aβ production in our L150P neurons. Moreover, *SORL1* has been proposed to influence Tau pathology, and *SORL1* SNPs have been linked to increased p-Tau levels (Elias-Sonnenschein et al., 2013). This aligns with the significant changes in p-Tau in L150P neurons. It is thus evident that the genetic background of our AD patients impacts their neuronal phenotypes, and other AD-related SNPs might contribute to differences in cellular phenotypes, providing additional AD risk or protective factors besides the presence of *PSEN1* mutations.

**FIGURE 6:**
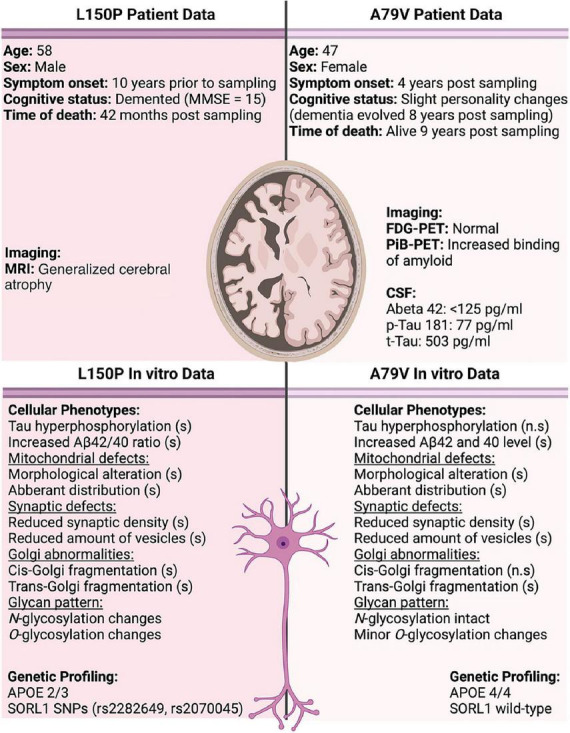
Overview of patient data compared to observations in our *in vitro* cell models. Significant changes are indicated by (s), and non-significant by (n.s). CSF normal reference values; Abeta 42 > 400 pg/ml, p-Tau 181 < 80 pg/ml, and *t*-Tau < 300 pg/ml.

Notably, the two patients in the study presented with clinical differences (Figure 6). The L150P carrier was a 58-year-old male, with established dementia at the time of sampling and generalized cerebral atrophy, identified through magnetic resonance imaging (MRI). The A79V carrier was a 48-year-old female displaying only slight personality changes, with dementia evolving eight years after sampling. An MRI was not performed for this patient. However, a fluorodeoxyglucose (FDG)-positron emission tomography (PET) scan was normal, whilst a Pittsburgh compound B-PET scan revealed increased cortical binding of amyloid in a pattern typical for fAD-linked *PSEN1* mutations. Pathological levels of Aβ and Tau were detected in her CSF.

*Presenilin 1 (PSEN1)* has been suggested to not only act as the catalytic subunit of γ-secretase, but also as a regulator of APP trafficking to the cell surface. fAD-linked mutations have been demonstrated to reduce this APP cell surface delivery, whereas loss-of-function mutations lead to APP accumulation at the plasma membrane (Cai et al., 2003). *PSEN1* mutations could thus modulate APP processing through multiple mechanisms. The different physical location of the A79V and L150P mutations within *PSEN1* could further contribute to the observed diverse phenotypic outcome. L150P is located closer to the catalytic protein domain, which could exacerbate the neuronal disease phenotype, whilst the A79V mutation is located at the intracellular C-terminal end.

In summary, our findings demonstrate that fAD hiPSC derived neurons display hallmark AD pathologies. We identify Golgi fragmentation as one of the earliest neuronal events in AD pathogenesis and provide insight of a causative mechanism of Aβ accumulation potentially in combination with Tau hyperphosphorylation. We show that glycosylation depends on multiple mechanisms and suggest that the *SORL1* genetic background and expression level could contribute to maintain Golgi functionality. The demonstration of Golgi-related phenotypes in additional model systems clearly indicates it as a universal perturbation in both familial early- and sporadic late-onset AD, validating the clinical relevance of our findings. Pharmaceutical intervention strategies targeting these early processes could be a potential new treatment avenue. Notably, long-term changes in the glycosylation profile could alter the overall cell surface composition, potentially inducing an inflammatory response. A remarkable future aspect of this study would therefore be evaluation of neuron-to-microglia interactions in co-culture systems. Moreover, the precise relationship and mechanism between *SORL1* and Golgi fragmentation remains to be fully elucidated and will thus be interesting to investigate further.

## Data availability statement

The datasets presented in this study can be found in online repositories. The names of the repository/repositories and accession number(s) can be found below: NCBI GEO database with the accession number GEO: GSE211993.

## Ethics statement

The studies involving human participants were reviewed and approved by Ethics Committee of the Capital Region of Denmark (H-4-2011-157). The patients/participants provided their written informed consent to participate in this study.

## Author contributions

KF conceived and designed the analysis. HH and KF performed the experimental design. HH performed most of the experiments. SV, PS, and FM performed the western blot analysis. MM performed the mesoscale analysis. PJ and ML performed the proteomics analysis. NH, FA, and HW performed the glycan profiling. GC, ND, VG, and JG performed the computational analyses of the RNA sequencing data. SK and DN were involved in assessment and Airyscan microscopy of N2A cells. SC was involved in the supervision of the work and contributed with the qPCR. AS, TN, and JN provided regulatory approvals, patients’ consent, and clinical data. AP and CP generated cell lines. AC, PH, BA, and RM contributed to the manuscript preparation. All authors read and approved the final manuscript.

## Footnotes

1
http://jgi.doe.gov/data-and-tools/bb-tools/
