# Supplementary material for: Golgi fragmentation – One of the earliest organelle phenotypes in Alzheimer’s disease neurons
Source: Front Neurosci. 2023 Feb 16;17:1120086. doi: 10.3389/fnins.2023.1120086 (PMC9978754; doi:10.3389/fnins.2023.1120086)
Supplement: Supplementary file 1 [file Data_Sheet_1.pdf]

Supplementary Material

Golgi fragmentation - One of the earliest organelle phenotypes in Alzheimer's disease neurons

SUPPLEMENTARY INFORMATION

1 Supplementary Figures

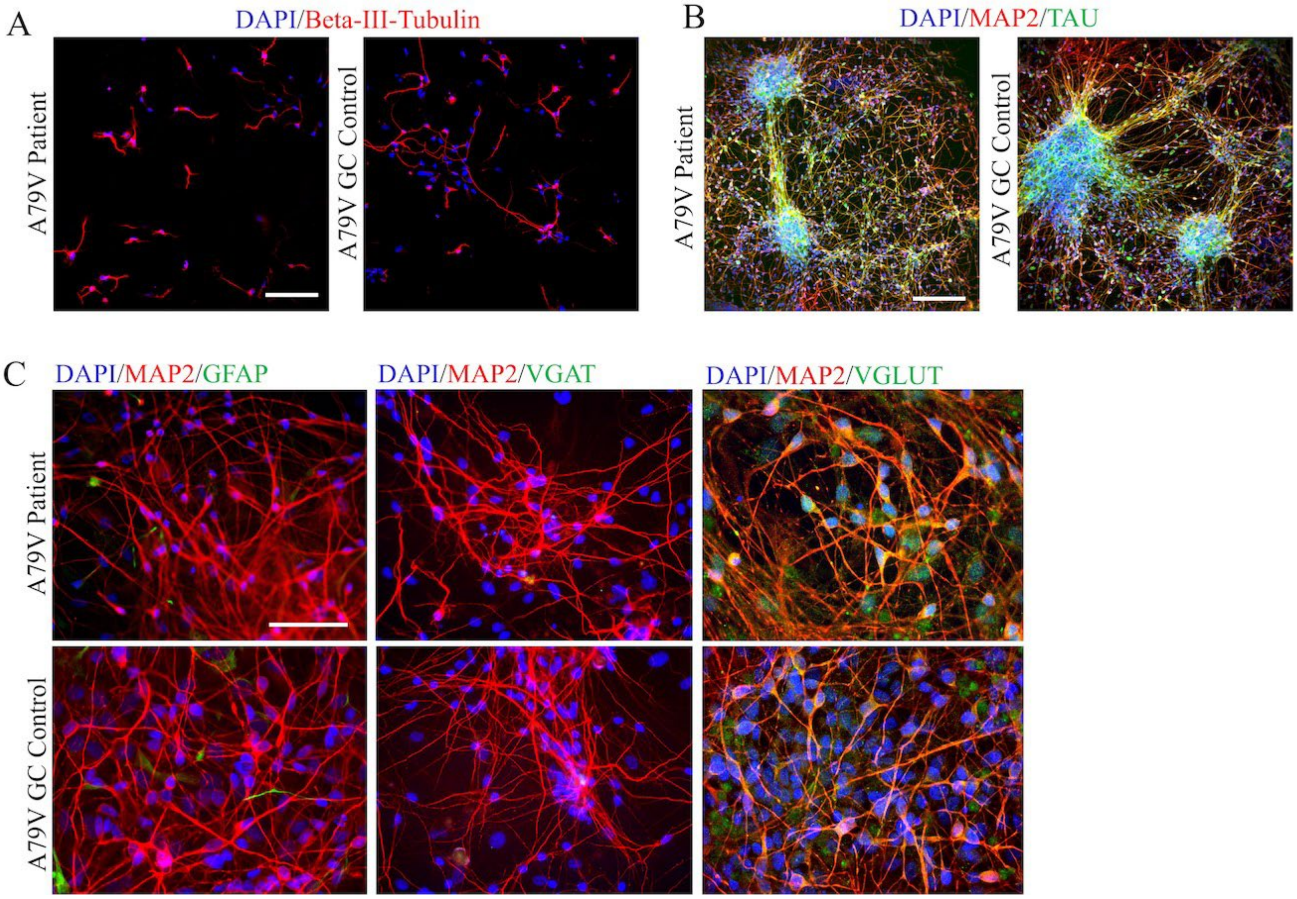

Figure S1. Generation and characterization of A79V hiPSC derived neurons. **A.** Neurite outgrowth analysis via ICC expression of Beta-III-tubulin. Scale bar 100 μm. **B.** Representative ICC images of neuronal markers MAP2 and TAU. Scale bar 100 μm. **C.** Representative ICC images of MAP2,

astrocytic marker GFAP, GABAergic neuron marker VGAT and glutamatergic neuron marker VGLUT. Scale bar 50 μm.

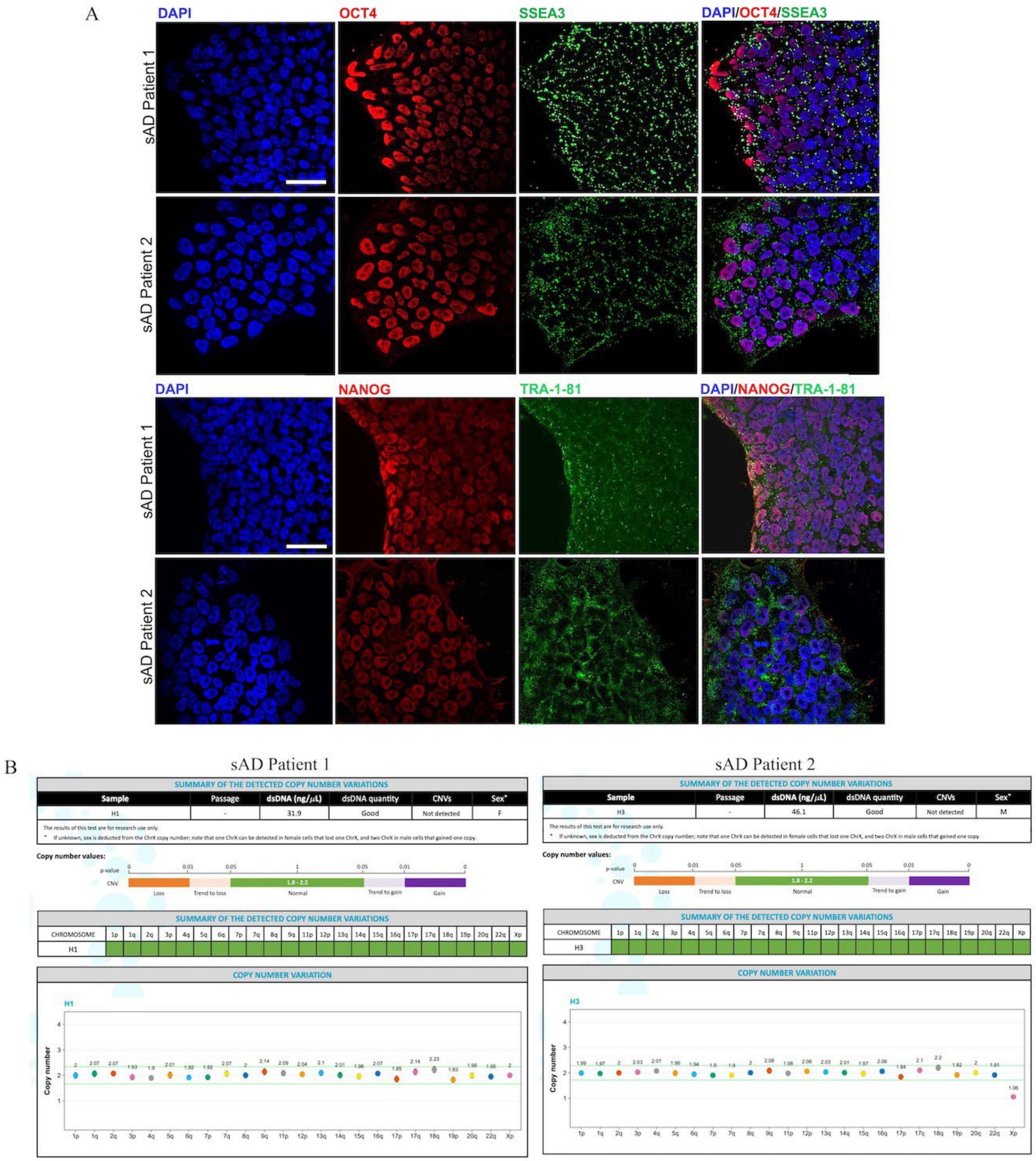

Figure S2. Generation, characterization and quality assessment of established sporadic AD hiPSC lines. **A.** Representative ICC images of pluripotency markers OCT4 and NANOG (red), as well as SSEA3 and TRA-1-81 (green). Scale bar 50 μm. **B.** Copy number variance (CNV) analysis.

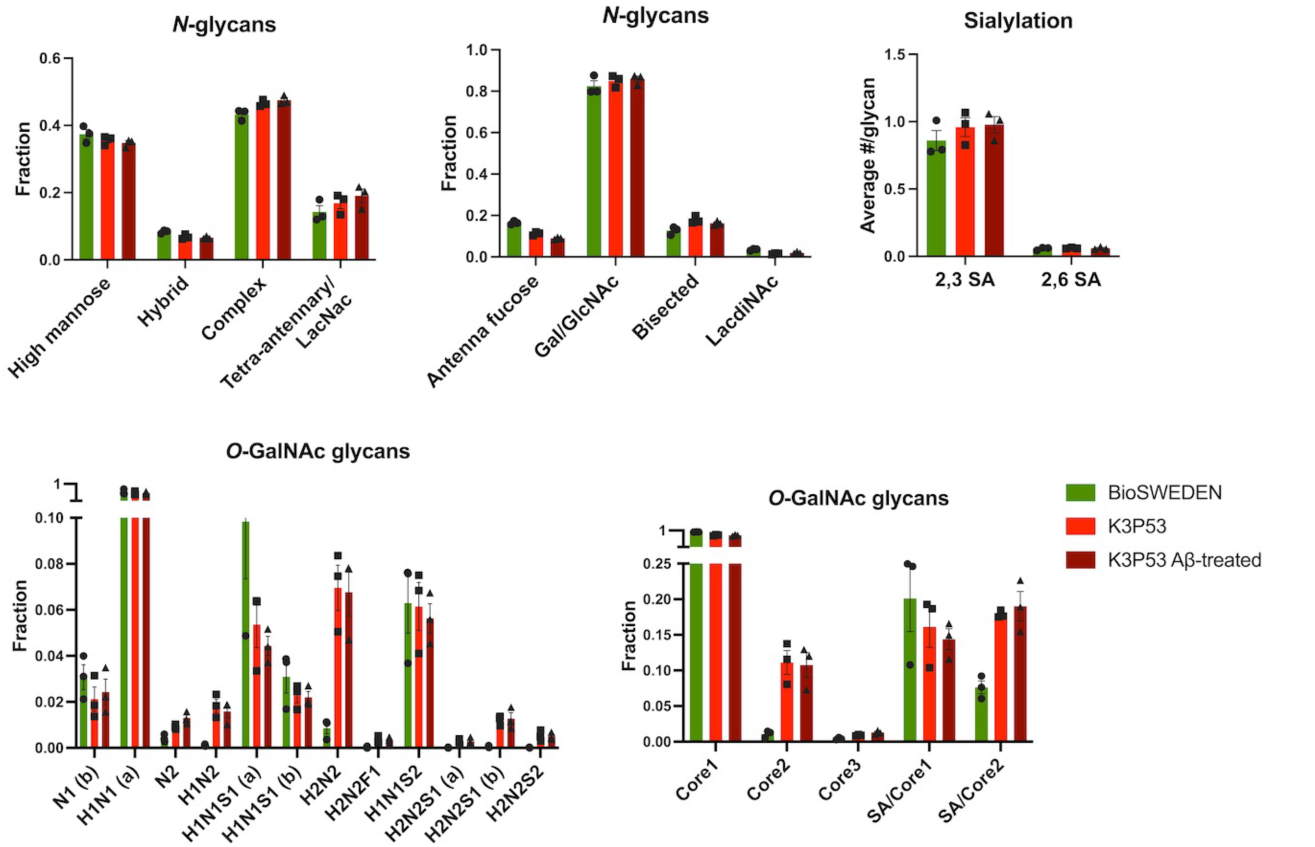

Figure S3. *N*- and *O*-glycan profiling of wild-type (K3P53), knock-in BioSweden and Aβ-treated wild-type hiPSC derived neurons.

2 Supplementary Tables

Table S1A. Summary of cell lines used in the study. Information on cell lines included in the study, displaying name, genotype, disease status, sex, age, karyotype, expression of pluripotency markers and publication reference.

hiPSC line	Genotype	Status	Sex	Age	Karyotype	Pluripotency associated marker	Reference
L150P	c.449 T>C	Symptomatic AD	Male	58	Normal	+	(Tubsuwan et al., 2016)
L150P GC	Gene correction	Isogenic control	Male	58	Normal	+	(Poon et al., 2016)
A79V	c.236 C>T	Pre-symptomatic AD	Female	48	Normal	+	(Li et al., 2016)
A79V GC	Gene correction	Isogenic control	Female	48	Normal	+	(Pires et al., 2016)
K3P53	Wild-type	Control	Male	18	Normal	+	(Rasmussen et al., 2014)
Bio SWEDEN	APPswe	Knock-in fAD	Male	18	Normal	+	(Frederiksen et al., 2019)

sAD Patient 1	-	sAD	Female	71	Normal	+	n/a
sAD Patient 2	-	sAD	Male	70	Normal	+	n/a

Table S1B. List of antibodies used for immunocytochemistry. Overview of antibodies used for the different immunocytochemistry assessments, including both primary and secondary antibodies, as well as information on dilution factors, company, cat. no. and RRID.

**Assessment**	**Antibody**	**Dilution**	**RRID**	**Company and Cat. No.**
iPSC verification	Goat anti-OCT4	1:500	AB_653551	Santa Cruz: Sc-8628
	Rabbit anti-NANOG	1:500	AB_1268451	PeproTech: 500-P236
	Rat anti-SSEA3	1:100	AB_1236554	Biolegend: 330302
	Mouse anti-SSEA4	1:100	AB_1089208	Biolegend: 330402
NPC verification	Mouse anti-NESTIN	1:500	AB_2251134	Millipore: MAB5326
	Rabbit anti-PAX6	1:100	AB_291612	Covance: PRB-278P
Neurite outgrowth	Mouse anti-TUJ1	1:100	AB_2210524	Millipore: MAB1637
Neuron verification	Mouse anti-MAP2	1:500	AB_477171	Sigma: M1406

	Rabbit anti-MAP2	1:500	AB_10807820	Millipore: AB2290
	Rabbit anti-TAU	1:200	AB_10013724	Dako: A0024
	Rabbit anti-VGLUT	1:500	AB_887875	Synaptic systems: 135303
	Rabbit anti-VGAT	1:500	AB_2301998	Millipore: AB5062P
	Rabbit anti-GFAP	1:500	AB_2109645	Millipore: AB5804
Golgi fragmentation	Sheep anti-TGN46	1:500	AB_324049	Bio-Rad: AHP500
	Mouse anti-GM130	1:500	AB_398141	BD Biosciences: 610822
	Mouse anti-γ-adaptin	1:500	AB_397768	BD Biosciences: 610385
Synaptic density	Rabbit anti-SY38	1:200	AB_2198854	Abcam: AB8049
Secondary antibodies	Alexa Fluor 488 donkey anti-mouse IgG	1:500	AB_141607	Thermo Fisher Scientific: A21202
	Alexa Fluor 488 donkey anti-rabbit IgG	1:500	AB_141708	Thermo Fisher Scientific: A21206
	Alexa Fluor 594 donkey anti-mouse IgG	1:500	AB_141633	Thermo Fisher Scientific: A21203
	Alexa Fluor 594 donkey anti-rabbit IgG	1:500	AB_141637	Thermo Fisher Scientific: A21207

	Alexa Fluor 594 donkey anti-rat IgG	1:500	AB_2535795	Thermo Fisher Scientific: A21209
	Alexa Fluor 594 donkey anti-goat IgG	1:500	AB_2534105	Thermo Fisher Scientific: A11058
	Alexa Fluor 594 donkey anti-sheep IgG	1:500	AB_2534083	Thermo Fisher Scientific: A11016

Table S1C. List of antibodies used for western blot. Overview of antibodies used for the Tau western blot assessment, including both primary and secondary antibodies, as well as information on dilution factors, company, cat. no. and RRID.

**Assessment**	**Antibody**	**Dilution**	**RRID**	**Company and Cat. No.**
Tau phosphorylation	Rabbit anti-phospho-tau Ser199/Ser202	1:1000	AB_2533749	Thermo Fisher Scientific: 44768G
	Mouse anti-PHF-Tau 181	1:500	AB_223651	Thermo Fisher Scientific: MN1050
	Rabbit anti-tau phospho S422	1:500	AB_1603345	Abcam: Ab79415
	Rabbit anti-phospho-tau Ser396	1:1000	AB_2533745	Thermo Fisher Scientific: 44752G

Normalization	Rabbit anti-GAPDH	1:5000	AB_10167668	Santa Cruz: sc-25778
	Mouse anti-β-actin	1:5000	AB_476744	Sigma: A5441
Secondary antibodies	IRDye 800CW donkey anti-mouse IgG	1:15000	AB_621847	LI-COR: 926-32212
	IRDye 680LT donkey anti-rabbit	1:15000	AB_10706167	LI-COR: 926-68023

Table S1D. List of selected differentially expressed genes identified by bioinformatic analyses of RNA sequencing. Overview of selected differentially expressed genes identified through RNA sequencing and bioinformatic analyses, including involved compartment/pathway, gene name and ensemble ID.

**Compartment/Pathway**	**Gene**	**Ensemble ID**
Mitochondria - oxidative stress	SCARA3	ENSG00000168077
Mitochondria - metabolism	ACSF2	ENSG00000167107
	NME4	ENSG00000103202
	ACSS1	ENSG00000154930
	ACSL6	ENSG00000164398
	DGAT2	ENSG00000062282

Mitochondria - ATP/energy production	ME1	ENSG00000065833
	ACADL	ENSG00000115361
	ACOT11	ENSG00000162390
	CKMT1B	ENSG00000237289
	CKMT1A	ENSG00000223572
Synapses - receptors	SNAP25	ENSG00000132639
	GRM7	ENSG00000196277
	GRIA1	ENSG00000155511
	SYAP1	ENSG00000169895
	SYP	ENSG00000102003
	GRIN1	ENSG00000176884
	CACNG5	ENSG00000075429
	CHRNA6	ENSG00000147434
	SHC4	ENSG00000185634
	UNC5C	ENSG00000182168

GABRA2	ENSG00000151834
GRIA4	ENSG00000152578
GABRA4	ENSG00000109158
GABRG2	ENSG00000113327
CHRNA4	ENSG00000101204
GLRA3	ENSG00000145451
CHRM2	ENSG00000181072
GAD1	ENSG00000128683
UNC13C	ENSG00000137766
CDH10	ENSG00000040731
SYNDIG1	ENSG00000101463
PPFIBP2	ENSG00000166387
LRRTM1	ENSG00000162951
PCLO	ENSG00000186472
DSCAM	ENSG00000171587
KCNJ3	ENSG00000162989

IL1RAPL1	ENSG00000169306
SLITRK2	ENSG00000185985
SLITRK1	ENSG00000178235
SLC8A3	ENSG00000100678
ASIC2	ENSG00000108684
TENM2	ENSG00000145934
SLC4A10	ENSG00000144290
SH3GL3	ENSG00000140600
SH3GL2	ENSG00000107295
CADPS2	ENSG00000081803
TANC1	ENSG00000115183
FAM107A	ENSG00000168309
NRN1	ENSG00000124785
PDLIM4	ENSG00000131435
SNCB	ENSG00000074317

Synapses - vesicles	SNCG	ENSG00000173267
	CACNA1A	ENSG00000141837
	SYNPO	ENSG00000171992
Synapses - transmission	SH2D5	ENSG00000189410
	KCNA3	ENSG00000177272
	KCNN2	ENSG00000080709
	HTR5A	ENSG00000157219
	SYNC	ENSG00000162520
	SV2C	ENSG00000122012
	SLC17A6	ENSG00000091664
	SLC6A17	ENSG00000197106
	WASF2	ENSG00000158195
	SYT7	ENSG00000011347
	SYT2	ENSG00000143858
	FRMPD4	ENSG00000169933
Golgi - glycosylation	GALNT9	ENSG00000182870

GALNTL6	ENSG00000174473
GALNT13	ENSG00000144278
BGN	ENSG00000182492
DCN	ENSG00000011465
MAN1C1	ENSG00000117643
XYLT2	ENSG00000015532
UGT8	ENSG00000174607
PLOD3	ENSG00000106397
MGAT4C	ENSG00000182050
MGAT3	ENSG00000128268
CHST9	ENSG00000154080
SDC4	ENSG00000124145
B4GALT1	ENSG00000086062
BCAN	ENSG00000132692
FUT1	ENSG00000174951

Golgi - organization/dynamics	OGN	ENSG00000106809
	HSPG2	ENSG00000142798
	A4GALT	ENSG00000128274
Golgi - transport	CLEC18B	ENSG00000140839
	FYCO1	ENSG00000163820
	GORASP1/2	ENSG00000114745
	GOLGA2/GM130	ENSG00000167110
	COPZ2	ENSG00000005243
	RAB13	ENSG00000143545
	RAB34	ENSG00000109113
	RAB11FIP5	ENSG00000135631
	RAB7B	ENSG00000276600
	PLIN3	ENSG00000105355
	SORL1	ENSG00000137642

Table S1E. List of primers used for qPCR. Overview of differentially expressed target genes validated by qPCR, including gene name and ensemble ID, as well as forward and reverse primer design.

Target Gene	Ensemble ID	Forward Primer	Reverse Primer
CKMT1A	ENSG00000223572	GATTCTGCCGAGGCCTCAAA	CCAGTGCCCAGGTTAGATGG
SCARA3	ENSG00000168077	CCGGAACCTCTCCATGATCG	GCCCTTTCACGCCCATATCT
FYCO1	ENSG00000163820	GGCCCCAGAAAGTTTCGGTT	CCTTGTTCTGTGGGCACTCT
GORASP	ENSG00000114745	AGGTCTGGGAAGGGGATATGA	TCCACAATCTCTCGATGCCG
GOLGA2	ENSG00000167110	CTAGCGGTAGCCCTGGACTC	CCCTGCTTTTGGTGGCATTC
SORL1	ENSG00000137642	GGATACGGACTGCCAGGATG	AGCAATCACGCAGACCATCA
SYNDIG1	ENSG00000101463	GCAGCCTTCTACTTGTCCCA	CACATAGACGCCAGTCCCAA
GRIA4	ENSG00000152578	AGGTGAATGTGGACCCAAGG	CAAGCCGCCAACCAGAATGT
SYT7	ENSG00000011347	ACGAAGGGGACCATGTACCG	CCGCAGAGGACGATAGTGAC
GAPDH	ENSG00000111640	CTCTCTGCTCCTCCTGTTCGAC	TGAGCGATGTGGCTCGGCT

Table S2A. Statistical analyses of observed cellular phenotypes in seven-week hiPSC derived neurons. Statistical analyses of observed cellular phenotypes in seven-week neurons, including western blot for Tau analysis, mesoscale for Aβ secretion, neurite outgrowth assessment, synaptic density assessment and mitochondria TEM morphometry analysis. Results are displayed as mean ± standard error of the mean (SEM) from three replicates.

Assessment	Cell line	Mean ± SEM (7 weeks)

Western blot (Ser199/Ser202)	L150P	3.179 ± 1.6
	L150P GC	1.108 ± 0.1253
	A79V	1.423 ± 0.6141
	A79V GC	0.0301 ± 0.01727
Western blot (T181)	L150P	3.462 ± 0.3366**
	L150P GC	0.9135 ± 0.1931
	A79V	0.036 ± 0.01069
	A79V GC	0.021 ± 0.01002
Western blot (S422)	L150P	2.718 ± 0.4762*
	L150P GC	0.3592 ± 0.1359
	A79V	2.28 ± 0.9796
	A79V GC	2.2 ± 1.113
Western blot (Ser396)	L150P	1.135 ± 0.203
	L150P GC	1.098 ± 0.3337
	A79V	2.98 ± 0.6145

	A79V GC	0.4967 ± 0.0865
Aβ40 (Mesoscale)	L150P	34.42 ± 1.334****
	L150P GC	54.34 ± 0.9868
	A79V	36.03 ± 1.164****
	A79V GC	15.13 ± 0.6698
	BioSWEDEN	92.99 ± 1.855****
	K3P53	202.5 ± 0.2531
Aβ42 (Mesoscale)	L150P	5.132 ± 0.1064****
	L150P GC	7.210 ± 0.02053
	A79V	6.946 ± 0.1206****
	A79V GC	2.767 ± 0.03049
	BioSWEDEN	13.30 ± 0.4567****
	K3P53	20.38 ± 0.1889
Aβ42/Aβ40 ratio	L150P	0.1497 ± 0.002891**
	L150P GC	0.1329 ± 0.002744

	A79V	0.1942 ± 0.009176
	A79V GC	0.1845 ± 0.007334
	BioSWEDEN	0.1428 ± 0.002615****
	K3P53	0.1006 ± 0.0008222
Neurite outgrowth (Length)	L150P	799.28 ± 126.53
	L150P GC	937.77 ± 135.48
	A79V	176.74 ± 26.7364
	A79V GC	196.82 ± 13.7152
Synaptic density (SY38 puncta)	L150P	23.47 ± 0.9735***
	L150P GC	29.47 ± 1.14
	A79V	14.96 ± 0.7703**
	A79V GC	19.43 ± 1.293
Total mitochondria (TEM)	L150P	0.0365 ± 0.002
	L150P GC	0.034 ± 0.0032
	A79V	0.0382 ± 0.002

	A79V GC	0.0308 ± 0.0051
Normal mitochondria (TEM)	L150P	0.01539 ± 0.0014
	L150P GC	0.01998 ± 0.002
	A79V	0.01846 ± 0.0023
	A79V GC	0.0192 ± 0.0037
Cristaeless mitochondria (TEM)	L150P	0.01077 ± 0.0011*
	L150P GC	0.00667 ± 0.0012
	A79V	0.01814 ± 0.0024*
	A79V GC	0.00856 ± 0.0023
Mitochondria size (TEM)	L150P	1.1921 ± 0.04695
	L150P GC	1.2459 ± 0.0518
	A79V	1.4373 ± 0.03689
	A79V GC	1.4199 ± 0.0466

Table S2B. Statistical analyses of mitochondria and Golgi alteration in five- and seven-week hiPSC derived neurons. Statistical analyses of mitochondria and Golgi alteration in five- and seven-week neurons. Results are displayed as mean ± standard error of the mean (SEM) from three replicates.

**Assessment**	**Cell line**	**Mean ± SEM (5 weeks)**	**Mean ± SEM (7 weeks)**
MitoTracker™	L150P	1472 ± 482.5	800.9 ± 233.3**
(Mean area of distribution)	L150P GC	2603 ± 1004	2249 ± 309.1
	A79V	1358 ± 110.9	808.4 ± 172.7*
	A79V GC	1285 ± 99.88	2671 ± 666.0
	BioSWEDEN	-	745.7 ± 55.06*
	K3P53	-	920.1 ± 54.97
Cis-Golgi surface area	L150P	256.5 ± 98.01	115 ± 12.00**
ICC (GM130)	L150P GC	150.4 ± 55.29	28 ± 7.9
	A79V	187.2 ± 31.73*	140.31 ± 13.65
	A79V GC	35.83 ± 4.550	108.9 ± 24.1
	BioSWEDEN	-	272.0 ± 29.92**
	K3P53	-	167.1 ± 13.88
	L150P GC (Aβ)	-	348.8 ± 51.89****

	A79V GC (Aβ)	-	152.0 ± 28.74
Trans-Golgi surface area	L150P	61.63 ± 7.473	36 ± 4.43*
ICC (TGN38)	L150P GC	35.97 ± 16.80	22 ± 0.50
	A79V	240.0 ± 40.49*	49.3 ± 8.2436*
	A79V GC	35.97 ± 16.80	25.56 ± 5.009
	BioSWEDEN	-	371.0 ± 34.25****
	K3P53	-	80.84 ± 5.848
	L150P GC (Aβ)	-	234.3 ± 24.02****
	A79V GC (Aβ)	-	118.0 ± 11.85****

Table S2C. RNA sequencing (RNA-seq) and proteomics statistics. RNA sequencing (RNA-seq) and proteomics statistical data. RNA-seq data is displayed as log2 fold change and adjusted p-value, and proteomic data is demonstrated by abundance ration and adjusted p-value. – indicates no measured expression.

Gene	Cell Line	RNA-seq analysis	RNA-seq analysis	Proteomics analysis	Proteomics analysis
		**Log2 Fold Change**	**Adjusted p-value**	**Abundance ratio**	**Adjusted p-value**
SCARA3	L150P	-2.43	9.89e-39	0.447	4.84e-07

	A79V	-2.19	6.93e-20	0.658	8.18e-06
ACSF2	L150P	-1.14	4.22e-24	0.79	0.0002
	A79V	-1.93	4.18e-51	0.669	7.27e-06
NME4	L150P	-1.18	5.34e-13	0.828	0.16
	A79V	-1.15	1.62e-18	0.788	0.02
ACSS1	L150P	-2.80	5.39e-24	1.311	0.0079
	A79V	-2.52	1.49e-15	0.562	2.28e-05
ACSL6	L150P	1.85	2.15e-25	1.645	1.19e-06
	A79V	1.797	5.23e-15	1.275	0.0001
DGAT2	L150P	1.17	2.66e-09	-	-
	A79V	1.29	4.75e-11	-	-
ME1	L150P	-1.02	8.53e-25	0.531	6.00e-06
	A79V	-1.81	6.01e-09	0.633	4.21e-05
ACADL	L150P	-2.12	8.01e-33	1.172	0.009
	A79V	1.65	7.30e-08	0.744	0.0001
ACOT11	L150P	1.02	0.015	-	-
	A79V	-1.55	0.0002	-	-

CKMT1B	L150P	2.004	1.03e-29	1.697	5.13e-07
	A79V	1.03	3.32e-09	1.122	0.002
CKMT1A	L150P	1.59	2.02e-18	1.697	5.13e-07
	A79V	1.01	1.127e-08	1.122	0.002
SNAP25	L150P	0.95	1.49e-46	1.661	2.94e-05
	A79V	0.72	0.001	1.154	0.01
GRM7	L150P	1.90	1.85e-120	2.31	4.74e-07
	A79V	0.20	0.35	1.278	0.0001
GRIA1	L150P	-1.03	4.06e-110	0.803	7.72e-06
	A79V	1.35	3.70e-55	1.272	4.70e-06
SYAP1	L150P	-0.24	0.03	1.08	0.02
	A79V	-0.21	0.03	0.992	0.97
SYP	L150P	0.61	5.54e-06	1.693	2.24e-05
	A79V	1.24	1.41e-09	1.247	0.001
GRIN1	L150P	1.10	4.70e-06	-	-
	A79V	0.82	0.0002	-	-
CACNG5	L150P	2.92	2.28e-150	-	-

	A79V	3.24	2.70e-42	-	-
CHRNA6	L150P	1.13	0.0001	-	-
	A79V	1.96	2.71e-25	-	-
SHC4	L150P	1.36	1.24e-32	-	-
	A79V	1.85	1.37e-24	-	-
UNC5C	L150P	-1.08	1.17e-29	1.052	1
	A79V	-1.77	6.70e-20	0.675	0.0001
GABRA2	L150P	1.35	9.87e-37	1.776	3.16e-06
	A79V	1.58	2.12e-19	1.135	0.01
GRIA4	L150P	1.05	1.12e-18	1.091	0.06
	A79V	1.58	8.92e-19	1.1	0.02
GABRA4	L150P	2.00	7.07e-36	-	-
	A79V	1.39	9.93e-12	-	-
GABRG2	L150P	1.74	1.92e-76	2.049	4.37e-07
	A79V	1.46	1.76e-11	1.339	2.66e-05
CHRNA4	L150P	1.16	2.71e-08	-	-
	A79V	1.39	6.49e-10	-	-

GLRA3	L150P	1.36	6.98e-16	-	-
	A79V	1.28	1.42e-09	-	-
CHRM2	L150P	3.46	1.25e-69	-	-
	A79V	1.17	2.83e-09	-	-
GAD1	L150P	1.69	1.74e-83	0.786	0.001
	A79V	1.38	2.13e-31	1.315	0.0007
UNC13C	L150P	1.54	2.59e-25	-	-
	A79V	1.26	2.55e-13	-	-
CDH10	L150P	1.29	2.86e-48	0.762	0.05
	A79V	1.51	1.70e-21	1.055	0.90
SYNDIG1	L150P	-1.28	7.60e-22	-	-
	A79V	-1.47	1.76e-19	-	-
PPFIBP2	L150P	1.30	4.43e-12	-	-
	A79V	-1.79	1.11e-16	-	-
LRRTM1	L150P	1.25	2.40e-28	-	-
	A79V	1.22	5.49e-14	-	-
PCLO	L150P	1.06	2.62e-13	-	-

	A79V	1.49	3.08e-19	-	-
DSCAM	L150P	1.55	9.75e-45	-	-
	A79V	1.02	3.99e-18	-	-
KCNJ3	L150P	1.80	5.60e-43	-	-
	A79V	1.69	7.01e-16	-	-
IL1RAPL 1	L150P	1.21	7.39e-23	-	-
	A79V	1.08	4.73e-12	-	-
SLITRK2	L150P	3.08	1.15e-221	1.567	3.48e-06
	A79V	1.03	1.61e-10	1.169	0.004
SLITRK1	L150P	1.45	1.44e-87	1.476	2.26e-06
	A79V	1.13	3.74e-09	1.215	0.0002
SLC8A3	L150P	1.56	4.63e-85	-	-
	A79V	1.16	2.54e-10	-	-
ASIC2	L150P	1.31	6.21e-09	-	-
	A79V	1.36	3.92e-10	-	-
TENM2	L150P	-1.24	1.09e-64	1.012	0.96
	A79V	1.20	8.59e-10	1.495	1.28e-06

SLC4A10	L150P	1.10	2.04e-14	1.019	0.33
	A79V	1.02	2.19e-09	1.121	0.0008
SH3GL3	L150P	1.08	2.14e-29	1.844	8.94e-07
	A79V	1.18	5.07e-09	1.117	0.02
SH3GL2	L150P	1.83	3.33e-178	2.123	4.849e-07
	A79V	1.16	5.79e-07	1.262	0.0003
CADPS2	L150P	2.78	3.26e-232	1.204	0.004
	A79V	-1.21	7.06e-09	2.264	1.15e-06
TANC1	L150P	-1.78	1.13e-105	-	-
	A79V	-1.009	2.03e-08	-	-
FAM107A	L150P	1.12	1.43e-24	-	-
	A79V	1.50	2.56e-08	-	-
NRN1	L150P	2.92	2.05e-161	2.211	5.503e-06
	A79V	1.05	2.62e-08	1.421	0.001
PDLIM4	L150P	-1.14	1.85e-05	0.338	1.56e-06
	A79V	-1.56	3.09e-08	0.456	1.82e-05
SNCB	L150P	1.23	3.30e-08	1.713	4.14e-05

	A79V	1.33	6.77e-08	1.211	0.03
SNCG	L150P	2.41	1.64e-05	1.617	7.65e-06
	A79V	1.01	0.002	1.243	0.001
CACNA1A	L150P	1.57	1.40e-37	-	-
	A79V	1.37	7.91e-08	-	-
SYNPO	L150P	-3.92	1.85e-185	0.427	5.13e-07
	A79V	-1.58	1.26e-06	0.712	7.76e-05
SH2D5	L150P	1.28	1.32e-07	-	-
	A79V	1.19	2.10e-05	-	-
KCNA3	L150P	1.15	1.68e-14	1.085	0.19
	A79V	1.02	2.90e-05	1.324	0.006
KCNN2	L150P	3.63	1.11e-29	-	-
	A79V	1.007	8.07e-05	-	-
HTR5A	L150P	1.39	0.0005	-	-
	A79V	1.15	0.0003	-	-
SYNC	L150P	-2.51	2.22e-249	0.496	2.00e-06
	A79V	-1.40	0.01	0.774	0.0006

SV2C	L150P	3.09	0.0	-	-
	A79V	-1.36	2.16e-19	-	-
SLC17A6	L150P	2.85	3.17e-146	2.896	6.21e-07
	A79V	1.44	1.64e-14	1.392	0.0006
SLC6A17	L150P	1.46	7.29e-22	1.559	5.98e-07
	A79V	1.47	4.13e-13	1.333	5.61e-06
WASF2	L150P	-1.26	6.29e-142	0.462	5.44e-07
	A79V	-1.24	2.84e-12	0.67	2.28e-05
SYT7	L150P	1.08	9.13e-17	2.257	1.98e-06
	A79V	1.06	1.98e-08	1.059	0.40
SYT2	L150P	3.46	1.93e-46	1.315	0.01
	A79V	2.26	3.05e-26	1.422	0.002
FRMPD4	L150P	1.26	8.34e-21	-	-
	A79V	1.20	1.94e-06	-	-
GALNT9	L150P	1.52	8.91e-07	-	-
	A79V	1.46	2.36e-11	-	-

GALNTL6	L150P	1.57	4.95e-40	-	-
	A79V	1.04	0.0002	-	-
GALNT13	L150P	1.96	3.21e-58	-	-
	A79V	1.13	1.49e-15	-	-
BGN	L150P	-2.82	4.96e-27	1.154	0.17
	A79V	-2.60	3.85e-121	0.408	4.16e-05
DCN	L150P	3.23	5.23e-65	1.143	0.37
	A79V	-3.19	4.62e-120	0.362	9.68e-05
MAN1C1	L150P	-1.88	1.14e-73	0.528	2.03e-07
	A79V	-1.45	1.00e-32	0.59	3.30e-07
XYLT2	L150P	-1.03	-4.72	1.092	0.43
	A79V	-1.29	4.51e-16	0.952	0.99
UGT8	L150P	2.14	9.46e-46	-	-
	A79V	-2.69	6.89e-16	-	-
PLOD3	L150P	-1.51	1.46e-12	0.54	4.07e-07
	A79V	-1.12	9.02e-13	0.592	5.77e-07
MGAT4C	L150P	1.54	2.75e-23	-	-

	A79V	1.65	1.14e-12	-	-
MGAT3	L150P	0.58	0.003	-	-
	A79V	0.24	0.04	-	-
CHST9	L150P	-1.43	7.22e-20	-	-
	A79V	1.07	6.92e-12	-	-
SDC4	L150P	-1.81	3.12e-77	1.286	0.07
	A79V	-1.97	2.11e-10	1.251	0.13
B4GALT1	L150P	-1.25	8.33e-42	-	-
	A79V	-1.70	4.87e-09	-	-
BCAN	L150P	1.34	1.56e-12	1.94	1.18e-05
	A79V	1.81	4.69e-08	1.512	0.0001
FUT1	L150P	1.37	1.004e-10	-	-
	A79V	1.40	3.873e-07	-	-
OGN	L150P	-1.22	1.58e-05	1.065	0.52
	A79V	-2.13	5.88e-07	0.422	1.91e-05
HSPG2	L150P	-1.68	1.46e-10	0.939	0.06
	A79V	-1.44	3.25e-06	0.87	0.002

A4GALT	L150P	-3.34	1.09e-12	-	-
	A79V	-2.58	9.15e-06	-	-
CLEC18B	L150P	-2.75	1.74e-32	0.791	0.01
	A79V	-1.57	0.009	0.931	0.27
FYCO1	L150P	-1.30	7.85e-51	0.795	0.0001
	A79V	-1.38	1.62e-18	0.754	3.24e-05
GORASP 1/2	L150P	-0.37	0.001	0.978	1
	A79V	-0.28	1.23e-07	1.002	1
GOLGA2/ GM130	L150P	-0.18	0.14	1.021	0.27
	A79V	-0.80	2.42e-56	0.82	1.15e-05
COPZ2	L150P	-2.30	1.05e-41	0.866	0.02
	A79V	-3.31	3.67e-78	1.375	0.50
RAB13	L150P	-1.01	8.52e-17	0.591	5.21e-05
	A79V	-1.58	1.66e-15	0.95	0.23
RAB34	L150P	-1.19	1.05e-18	0.61	4.53e-06
	A79V	-1.79	3.94e-15	0.519	1.13e-06

RAB11FIP5	L150P	-1.01	3.34e-09	0.961	0.87
	A79V	-1.04	3.87e-14	1.178	0.01
RAB7B	L150P	-1.69	1.66e-19	-	-
	A79V	-1.63	2.93e-13	-	-
PLIN3	L150P	-1.30	1.17e-12	0.536	1.22e-06
	A79V	-1.13	1.82e-08	0.411	5.34e-07
SORL1	L150P	0.48	8.98e-13	1.042	0.69
	A79V	1.26	8.50e-19	1.05	0.195
